# Identification of Novel Pathogenicity Loci in *Clostridium perfringens* Strains That Cause Avian Necrotic Enteritis

**DOI:** 10.1371/journal.pone.0010795

**Published:** 2010-05-24

**Authors:** Dion Lepp, Bryan Roxas, Valeria R. Parreira, Pradeep R. Marri, Everett L. Rosey, Joshua Gong, J. Glenn Songer, Gayatri Vedantam, John F. Prescott

**Affiliations:** 1 Department of Pathobiology, University of Guelph, Guelph, Ontario, Canada; 2 Guelph Food Research Centre, Agriculture and Agri-Food Canada, Guelph, Ontario, Canada; 3 Department of Veterinary Science and Microbiology, University of Arizona, Tucson, Arizona, United States of America; 4 BIO5 Institute, University of Arizona, Tucson, Arizona, United States of America; 5 Veterinary Medicine Research and Development, Pfizer Animal Health, Kalamazoo, Michigan, United States of America; 6 Southern Arizona VA Healthcare System, Tucson, Arizona, United States of America; Max Planck Institute for Infection Biology, Germany

## Abstract

Type A *Clostridium perfringens* causes poultry necrotic enteritis (NE), an enteric disease of considerable economic importance, yet can also exist as a member of the normal intestinal microbiota. A recently discovered pore-forming toxin, NetB, is associated with pathogenesis in most, but not all, NE isolates. This finding suggested that NE-causing strains may possess other virulence gene(s) not present in commensal type A isolates. We used high-throughput sequencing (HTS) technologies to generate draft genome sequences of seven unrelated *C. perfringens* poultry NE isolates and one isolate from a healthy bird, and identified additional novel NE-associated genes by comparison with nine publicly available reference genomes. Thirty-one open reading frames (ORFs) were unique to all NE strains and formed the basis for three highly conserved NE-associated loci that we designated NELoc-1 (42 kb), NELoc-2 (11.2 kb) and NELoc-3 (5.6 kb). The largest locus, NELoc-1, consisted of *netB* and 36 additional genes, including those predicted to encode two leukocidins, an internalin-like protein and a ricin-domain protein. Pulsed-field gel electrophoresis (PFGE) and Southern blotting revealed that the NE strains each carried 2 to 5 large plasmids, and that NELoc-1 and -3 were localized on distinct plasmids of sizes ∼85 and ∼70 kb, respectively. Sequencing of the regions flanking these loci revealed similarity to previously characterized conjugative plasmids of *C. perfringens*. These results provide significant insight into the pathogenetic basis of poultry NE and are the first to demonstrate that *netB* resides in a large, plasmid-encoded locus. Our findings strongly suggest that poultry NE is caused by several novel virulence factors, whose genes are clustered on discrete pathogenicity loci, some of which are plasmid-borne.

## Introduction

Avian necrotic enteritis (NE) is caused by specific strains of *Clostridium perfringens* and costs the worldwide poultry industry an estimated $2 billion annually, largely due to costs of antimicrobial prophylaxis and inefficient feed conversion [1], [2]. Elimination of routine antibiotic use (e.g., in the European Union) has been associated with increased incidence of NE. Considerable effort is being invested in research on both pathogenesis as well as immunity to infection as a basis for development of alternate control methods. The alpha toxin (CPA) of *C. perfringens* was long assumed to play a central role in the pathogenesis of NE, but Keyburn and others [3] showed that challenge of broiler chicks with a CPA mutant yielded lesions comparable to those in birds inoculated with the wildtype strain. In counterpoint, other studies have revealed that immunization of birds with CPA toxoid protects against virulent *C. perfringens* challenge [4], [5]. Furthermore, the intestines of birds challenged with the CPA mutant contained CPA [6], likely produced by the resident *C. perfringens* strains and contributing to gut lesions. Subsequently, a new toxin, NetB, was discovered that is related to the pore-forming alpha hemolysin of *Staphylococcus aureus* and beta toxin of *C. perfringens*. NetB is common in strains originating from chickens with NE and in those strains, is essential for NE development [7]. Recent work has largely confirmed the critical association of *netB* with NE strains [8], although *netB*-negative *C. perfringens* may also sometimes produce NE [9].

Comparative genomics is one approach to determine whether *C. perfringens* isolates from chickens with NE have a genetic “signature” distinct from either commensal strains, or those causing enteric disease in other hosts. Complete genome sequences are available for a gas gangrene isolate, a soil isolate, and a human food-poisoning isolate of *C. perfringens* [10], [11]. Comparison of the three genomes revealed considerable genomic diversity, with more than 300 unique “genomic islands”; furthermore, there is evidence of even greater diversity in other strains [11]. The genetic features in these islands may contribute to the varied disease manifestations that are characteristic of the diversity of *C. perfringens* as a pathogen [12]. Much of the virulence of toxinotypes A–E of *C. perfringens* depends upon genes harboured on large plasmids. The enterotoxin gene (*cpe*) may sometimes be plasmid-borne, and genes for beta2 toxin (*cpb2*) and other potential virulence determinants [13]–[17] are also found on large plasmids currently being characterized [17].

In this study, we sequenced multiple *C. perfringens* NE isolates, and used comparative genomics to identify NE-specific DNA sequences. We found both plasmid-borne and genomic loci containing multiple genes whose predicted products shared high similarity to bacterial virulence and virulence-associated factors.

## Results

### Sequencing and *de novo* assembly of *C. perfringens* genomes

Draft genome sequences for eight type A *C. perfringens* poultry isolates were generated via 454 and Illumina/Solexa sequencing technologies. Seven of these strains (CP4, JGS4143, JGS4140, JGS1521, JGS5252, JGS5621, JGS1651) were recovered from NE field cases from Canada and the US, and one (JGS1473) from the intestinal tract of a healthy bird (Table 1). The virulence of each NE isolate was subsequently confirmed in an experimental model (data not shown); two strains, CP4 and JGS4143, have been more extensively characterized [4], [9], [18]–[22]. Antibiotic resistance profiles of all strains revealed variable resistance to bacitracin and tetracycline (Table 1).

**Table 1:** List of *Clostridium perfringens* strains used in this study.

Strain	Type	Source	Associated Disease	NE virulence^1^	Antibiotic resistance^2^	Reference
CP4	A	Chicken	NE	++++	Tc	[18]
JGS4143	A	Chicken	NE	+++	-	[22]
JGS4140	A	Chicken	NE	++++	Tc, Bac	This study
JGS1521	A	Chicken	NE	N/A	Bac	This study
JGS5252	A	Chicken	NE	++	-	This study
JGS5621	A	Chicken	NE	+	Tc, Bac	This study
JGS1651	A	Chicken	NE	++++	Tc, Bac	This study
CP1	A	Chicken	NE	+++	Tc, Bac	[18]
CP2	A	Chicken	NE	++	Tc, Bac	[18]
CP3	A	Chicken	NE	++	Tc, Bac	[18]
CP6	A	Chicken	NE	−	-	[18]
JGS1473	A	Chicken	Healthy	−	Bac	This study
ATCC 13124	A	Human	Gas gangrene	ND^3^	ND	[80]
SM101	A	Human	Food-poisoning	ND	ND	[80]
Str13	A	Dog		ND	ND	[10]
NCTC 8239	A	Human	Food-poisoning	ND	ND	
F4969	A		Non-food-borne disease	ND	ND	[47]
ATCC 3626	B	Lamb		ND	ND	
JGS1495	C	Pig	Diarrhoea	ND	ND	
JGS1721	D	Sheep	Enteritis	ND	ND	
JGS1987	E	Cow	Enteritis	ND	ND	

1 Virulence is based on the NE scoring system described in [4].

2 Strains were tested for resistance to ampicillin, bacitracin (Bac), chloramphenicol, clindamycin, erythromycin, nalidixic acid, teicoplanin, tetracycline (Tc) and vancomycin.

3 ND, not determined. Strains CP4, JGS4143, JGS4140, JGS1521, JGS5252, JGS5621, JGS1651 and JGS1473 were sequenced in this study. All of the NE isolates are netB-positive, with the exception of CP6; although this strain was originally isolated from a chicken afflicted with NE, it was subsequently found to be avirulent.

The Roche 454 GS FLX system was used to generate sequences for seven of the strains, which were then assembled with the Newbler assembler (Roche). One strain (CP4) was sequenced in a single-end (2 lanes) and a paired-end (1 lane) run with the Illumina/Solexa Genome Analyzer, and the reads were assembled *de novo* with Velvet [23]. Both sequencing approaches yielded assemblies of similar quality (Table 2). The 454 sequencing runs generated average coverages ranging from 13.3× to 32.7× based on an estimated 3.2 Mb genome, while the combination of Solexa runs resulted in 262× coverage. The total number of contigs and N50 values associated with these draft genomes were comparable to that of the six publicly available draft *C. perfringens* genomes generated via a traditional WGS sequencing approach (Table 2).

**Table 2:** Summary of *C. perfringens* genome sequences used in this study.

Strain	Sequence Type	Total reads	Avg read length (nt)	N50 (bp)	Size (Mb)	Contigs	Proteins	Accn No.	Reference
CP4	Illumina Solexa GA	24,264,136^1^	35	83,876	3.6	99	3,489	-	This study
JGS4143	Roche 454	772,463	114	17,408	3.3	466	3,420	-	This study
JGS4140	Roche 454	243,977	390	170,860	3.7	111	3,368	-	This study
JGS1521	Roche 454	160,442	265	2,928	3.1	1,469	3,618	-	This study
JGS5252	Roche 454	288,622	283	57,941	3.2	135	3,061	-	This study
JGS5621	Roche 454	229,633	311	144,918	3.6	124	3,622	-	This study
JGS1651	Roche 454	270,702	386	163,406	3.8	124	3,497	-	This study
JGS1473	Roche 454	228,810	278	49,191	3.4	148	3,236	-	This study
ATCC 13124	Finished	-2	-	-	3.3	-	2,876	CP000246	[80]
SM101	Finished	-	-	-	2.9	-	2,558	CP000312	[80]
Str13	Finished	-	-	-	3	-	2,660	BA000016	[10]
NCTC 8239	WGS^3^	-	-	134,604	3.3	55	2,933	ABDY00000000	
F4969	WGS	-	-	96,499	3.5	74	3,197	ABDX00000000	
ATCC 3626	WGS	-	-	88,742	3.9	98	3,623	ABDV00000000	
JGS1495	WGS	-	-	117,588	3.7	84	3,354	ABDU00000000	
JGS1721	WGS	-	-	80,514	4.1	221	3,345	ABOO00000000	
JGS1987	WGS	-	-	88,895	4.1	101	3,586	ABDW00000000	

1 total reads in single-end (2 lanes) and paired end (1 lane) runs.

2 not applicable.

3 WGS, Whole genome shotgun.

Optical mapping was performed on CP4 genomic DNA to provide independent verification of the assembly and assist in contig scaffolding. The CP4 genome size estimated from the optical map was 3.4 Mb, while the combined length of the contigs aligned to the map was 3.26 Mb, indicating that ∼96% of the genome was correctly accounted for in this draft (Figure S1). In addition, ∼375 kb of sequence not aligned to the optical map, distributed into 37 contigs, was presumably composed of mainly extrachromosomal sequences.

### Identification of NE-associated loci

We used two complementary approaches to identify and confirm sequences that were both conserved among the various NE strains but absent or significantly divergent from the nine publicly available *C. perfringens* strains (ATCC 13124, SM101, Str. 13, NCTC 8239, F4969, ATCC 3626, JGS1495, JGS1721, JGS1987) (Table 1). In the first approach, the sequence alignment algorithm Mummer [24] was used to identify sequences unique to each NE strain by aligning the draft genomes of CP4 and JGS4143 with the nine reference genomes. Unique sequences from the two NE strains were then aligned with each other to identify those conserved between them, and the ORFs present in these unique regions were retrieved. CP4 and JGS4143 were found to contain 435 and 173 genes not found in any of the reference strains, of which 31 were common to both NE strains. The novelty of these ORFs among the NE strains was further confirmed using OrthoMCL [25], which predicts orthologs based on clustering of reciprocal best BLAST hits.

In the second approach, the Rapid Annotation using Subsystem Technology (RAST) system was used to identify NE-specific sequences [26]. The *C. perfringens* draft genome sequences were uploaded to the RAST server and automatic annotation was performed. GenBank files of the nine publicly-available *C. perfringens* genomes were also uploaded using original gene coordinates and names. RAST's sequence-based comparison tool was used to compare NE strain JGS4140 to all other *C. perfringens* strains. Protein identity values from the comparison were tabulated and genes conserved in the NE strains (>95%) but not in the non-NE strains (<90%) were filtered into a new table. Sequences of contigs containing NE-specific genes were further analysed using the BLAST algorithm [27] to confirm uniqueness to the NE strains.

**Figure 1:**
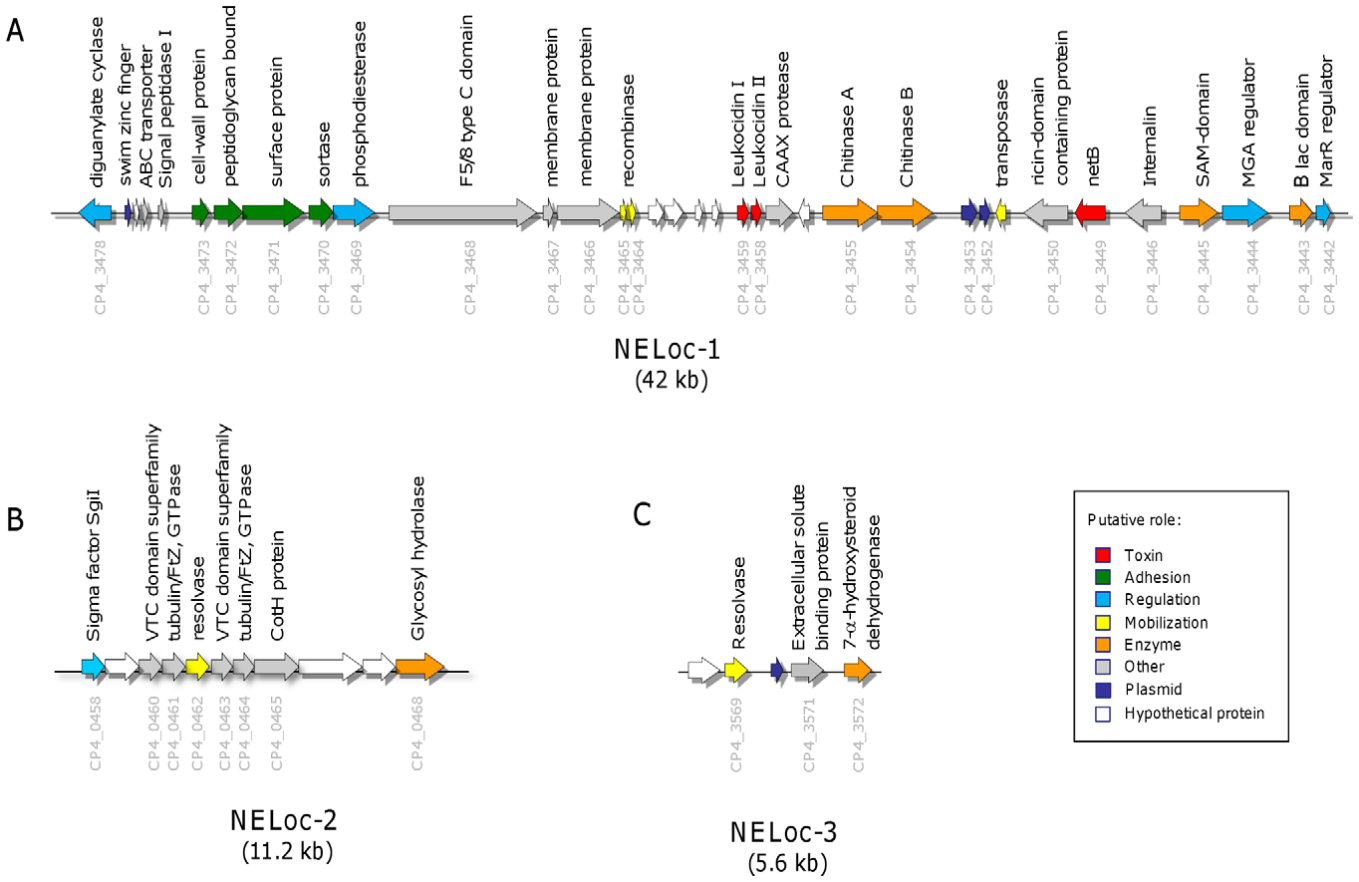
Genetic organization of NE-specific loci. The genetic organization of (A) NELoc-1, (B) NELoc-2 and (C) NELoc-3 is shown, each arrow representing a predicted gene and the total size given below each locus. Predicted functional annotations and locus tags are shown above and below each gene, respectively. Genes are colour-coded by their putative role based upon sequence analyses.

The majority of NE-specific genes identified using the methodology above were clustered on a small number of contigs, indicating that these genes were not dispersed randomly throughout the genome. A hybrid assembly was thus generated using the contigs from multiple NE strains to determine their absolute location and contiguity, and this revealed three unique DNA segments. First, a 46.6 kb scaffold was generated by this approach consisting of 37 genes, 25 of which had no predicted orthologs in the nine reference genomes, as determined by OrthoMCL (Table 3). The integrity of this scaffold was experimentally verified in two strains, CP4 and JGS4140, by closing all gaps and verifying anomalous regions through sequencing of PCR products. This resolved a novel ∼42 kb “pathogenicity” locus, which we designated NE locus 1 (NELoc-1). This locus harboured *netB*, as well as genes for other predicted proteins likely involved in virulence (Figure 1A and described below).

**Table 3:** Summary of predicted genes in NE pathogenicity locus NELoc-1.

Locus_tag^1^	Length (aa)	Predicted Product	Hit Description	E-value	% Identity^2^	Subcellular localization^3^	Conserved domains
CP4_3442*	148	transcriptional regulator, MarR family	transcriptional regulator, MarR family protein (Burkholderia thailandensis TXDOH)	4E-28	37.5% (136/150 aa)	Unknown (N)	HTH transcriptional regulator, MarR
CP4_3443*	249	beta-lactamase domain-containing protein	beta-lactamase domain-containing protein (Clostridium beijerinckii NCIMB 8052)	2E-94	67% (249/249 aa)	Unknown (N)	Lactamase_B; Metallo-hydrolase/oxidoreductase
CP4_3444*	496	M protein trans-acting positive regulator (MGA)	conserved hypothetical protein (Clostridium perfringens B str. ATCC 3626)	2E-79	41.8% (488/488 aa)	Cytoplasmic Membrane (N)	M trans-acting positive regulator
CP4_3445	418	putative radical SAM domain-containing protein	radical SAM domain protein (Clostridium perfringens B str. ATCC 3626)	0	89.7% (418/418 aa)	Unknown (N)	MoaA/nifB/pqqE, iron-sulphur binding, conserved site; Radical SAM
CP4_3446	391	putative internalin	putative Phosphoprotein phosphatase (Candidatus Cloacamonas acidaminovorans)	4E-41	42.1% (261/3445 aa)	Cellwall (Y)	Leucine-rich repeat; Immunoglobulin E-set:Toll-like receptor, leucine rich repeat
CP4_3447*	47	hypothetical protein	No significant hits				
CP4_3448*	42	hypothetical protein	No significant hits				
CP4_3449	322	necrotic enteritis toxin B	NetB (Clostridium perfringens)	0	99.6% (322/322 aa)	Extracellular (Y)	Leukocidin/haemolysin; Bi-component toxin, staphylococci; Leukocidin/porin
CP4_3450*	391	ricin-type beta-trefoil domain protein	hypothetical protein bthur0009_56310 (Bacillus thuringiensis serovar andalousiensis BGSC 4AW1)	8E-23	32.7% (290/1257 aa)	Unknown (N)	Ricin B lectin; Ricin B-related lectin
CP4_3451	93	transposase for transposon	conserved hypothetical protein (Clostridium perfringens D str. JGS1721)	7E-30	75.2% (93/271 aa)	Unknown (N)	Transposase, Tn3
CP4_3452	110	hypothetical protein	conserved hypothetical protein (Clostridium perfringens C str. JGS1495)	1E-50	94.5% (110/285 aa)	Cytoplasmic (N)	Conserved hypothetical protein CHP01784
CP4_3453	160	hypothetical protein	conserved hypothetical protein (Clostridium perfringens D str. JGS1721)	3E-85	97.5% (160/290 aa)	Unknown (N)	Conserved hypothetical protein CHP01784
CP4_3454*	599	chitinase B	chitinase B (Clostridium paraputrificum)	0	87.6% (598/831 aa)	Unknown (N)	Glycoside hydrolase, family 18, catalytic domain;Chitinase II;Glycoside hydrolase, catalytic core
CP4_3455*	611	chitinase A	glycosyl hydrolase, family 18 (Clostridium botulinum A2 str. Kyoto)	0	75.2% (613/617 aa)	Unknown (Y)	Glycoside hydrolase, family 18, catalytic domain;Glycoside hydrolase, chitinase active site;Carbohydrate-binding;Chitinase II:Glycoside hydrolase, catalytic core
CP4_3456*	106	hypothetical protein	hypothetical protein CD1711 (Clostridium difficile 630)	1E-30	60% (105/112 aa)	Cytoplasmic (N)	Uncharacterised protein family UPF0145
CP4_3457*	284	CAAX amino terminal protease family	CAAX amino terminal protease family (Fusobacterium nucleatum subsp. vincentii ATCC 49256)	6E-49	41.2% (286/293 aa)	Cytoplasmic Membrane (Y)	Abortive infection protein
CP4_3458*	96	leukocidin II	necrotic enteritis toxin B precursor (Clostridium perfringens)	4E-21	50% (94/322 aa)	Extracellular (N)	Leukocidin/haemolysin;Leukocidin/porin
CP4_3459*	114	leukocidin I	NetB (Clostridium perfringens)	2E-20	44.2% (113/322 aa)	Unknown (N)	Leukocidin/haemolysin;Leukocidin/porin
CP4_3460*	58	hypothetical protein	No significant hits				
CP4_3461*	66	conserved hypothetical protein	conserved hypothetical protein (Clostridium perfringens E str. JGS1987)	7E-08	63.4% (41/162 aa)	Unknown (N)	
CP4_3462*	200	conserved hypothetical protein	conserved hypothetical protein (Clostridium perfringens E str. JGS1987)	3E-99	88% (200/200 aa)	Unknown (N)	Flavoproteins
CP4_3463*	170	conserved hypothetical protein	conserved hypothetical protein (Clostridium perfringens E str. JGS1987)	4E-82	82.9% (170/170 aa)	Cytoplasmic (N)	Flavoproteins
CP4_3464*	38	resolvase/recombinase	resolvase/recombinase (Clostridium perfringens D str. JGS1721)	4E-13	100% (38/207 aa)	Extracellular (N)	
CP4_3465*	73	resolvase/recombinase	resolvase/recombinase (Clostridium perfringens D str. JGS1721)	1E-26	93.7% (64/207 aa)	Cytoplasmic (N)	Resolvase, N-terminal
CP4_3466	700	putative membrane protein	putative membrane protein (Clostridium perfringens NCTC 8239)	0	88.4% (699/851 aa)	Cytoplasmic Membrane (N)	Protein of unknown function DUF470;Protein of unknown function DUF471;Protein of unknown function DUF472
CP4_3467*	102	putative membrane protein	lysyl transferase (Clostridium perfringens C str. JGS1495)	2E-44	91.1% (102/851 aa)	Cytoplasmic Membrane (N)	
CP4_3468	1645	F5/8 type C domain-containing protein	hypothetical protein CPE1281 (Clostridium perfringens str. 13)	0	64.5% (1521/1687 aa)	Extracellular (Y)	Coagulation factor 5/8 type, C-terminal;Extracellular matrix-binding protein, Ebh;Galactose-binding like;Glycosyl hydrolase family 98, putative carbohydrate-binding module
CP4_3469*	450	diguanylate cyclase/phosphor-diesterase domain 2	diguanylate cyclase/phosphodiesterase domain 2 (Clostridium botulinum E1 str. ‘BoNT E Beluga’)	4E-88	43.6% (424/785 aa)	Cytoplasmic (N)	Diguanylate cyclase, predicted;Diguanylate phosphodiesterase, predicted
CP4_3470	265	sortase family protein	sortase family protein (Clostridium perfringens C str. JGS1495)	1E-104	81.4% (264/264 aa)	Unknown (Y)	Peptidase C60, sortase A/B
CP4_3471	677	putative surface protein	probable surface protein (Clostridium perfringens C str. JGS1495)	0	66.8% (486/519 aa)	Cellwall (LPXTG) (Y)	Surface protein from Gram-positive cocci;Collagen-binding surface protein Cna-like, B region
CP4_3472	365	peptidoglycan bound protein	cna protein B-type domain (Clostridium perfringens D str. JGS1721)	2E-96	76.1% (235/708 aa)	Unknown (N)	Collagen-binding surface protein Cna-like, B region
CP4_3473*	172	cell wall surface anchor family protein	cell wall surface anchor family protein (Clostridium perfringens C str. JGS1495)	5E-64	72.8% (166/757 aa)	Unknown (Y)	
CP4_3474*	44	signal peptidase I	signal peptidase I (Clostridium perfringens E str. JGS1987)	3E-10	80.4% (41/175 aa)	Extracellular (N)	
CP4_3475*	81	ABC transporter	putative ABC transporter, permease protein (Clostridium perfringens D str. JGS1721)	3E-27	76.5% (81/681 aa)	Unknown (N)	
CP4_3476*	48	hypothetical protein	No significant hits				
CP4_3477	54	swim zinc finger domain protein	swim zinc finger domain protein (Clostridium perfringens B str. ATCC 3626)	4E-21	94.2% (52/468 aa)	Unknown (N)	Zinc finger, SWIM-type
CP4_3478*	350	diguanylate cyclase/phosphor-diesterase	putative signaling protein (Clostridium hathewayi DSM 13479)	9E-27	40.6% (165/1261 aa)	Cytoplasmic Membrane (N)	Diguanylate cyclase, predicted

1 Based on strain CP4 genome. Genes for which an equivalent could not be identified in the nine sequenced reference genomes by OrthoMCL are indicated with an asterisk.

2 Percent amino acid identity (HSP length/total length of the subject protein).

3 Subcellular location as predicted by pSortb and, in brackets, whether a signal peptide was predicted by SignalP.

Second, another locus of 11.2 kb, NELoc-2 (Table 4; Figure 1B) was identified that consisted of 11 contiguous genes that were absent from eight of the nine reference genomes. This locus was found intact on contigs ranging in size from 22 kb–248 kb in five of the NE strains, while in the other three strains, it was separated into multiple contigs separated by gaps of 52 bp–162 bp, based on the sequence of strain CP4. This locus was also found in the non-NE, ovine enteritis strain JGS1721, in contigs of 11 kb and 120 kb. NELoc-2 shared >99.8% nucleotide identity among all NE strains while that of the JGS1721 strain was 99.1% identical.

**Table 4 pone-0010795-t004:** Summary of predicted genes in NE pathogenicity locus 2.

Locus_tag^1^	Length (aa)	Predicted Product	Hit Description	E-value	% Identity	Subcellular localization	Conserved domains
CP4_0458	216	sigma factor SgiI	putative DNA-directed RNA polymerase sigma factor (Clostridium perfringens D str. JGS1721)	1E-113	97.7% (215/219 aa)	Unknown (N)	RNA polymerase sigma factor, region 2;RNA polymerase sigma-70 region 2
CP4_0459	348	conserved hypothetical protein	hypothetical protein CJD_0460 (Clostridium perfringens D str. JGS1721)	1E-160	94.4% (337/348 aa)	Cellwall (N)	
CP4_0460	232	putative VTC domain superfamily	conserved hypothetical protein (Clostridium perfringens D str. JGS1721)	1E-129	99.6% (232/232 aa)	Unknown (N)	VTC domain
CP4_0461	226	putative tubulin/FtsZ, GTPase	conserved hypothetical protein (Clostridium perfringens D str. JGS1721)	1E-122	100% (226/226 aa)	Cytoplasmic Membrane (N)	
CP4_0462	183	resolvase	resolvase domain-containing protein (Methanococcus vannielii SB)	3E-57	57.1% (182/199 aa)	Unknown (N)	Resolvase, N-terminal;Recombinase, conserved site;Resolvase, helix-turn-helix region
CP4_0463	230	putative VTC domain superfamily	conserved hypothetical protein (Clostridium perfringens D str. JGS1721)	1E-130	99.6% (230/230 aa)	Unknown (N)	VTC domain
CP4_0464	222	tubulin/FtsZ, GTPase	tubulin/FtsZ, GTPase (Clostridium perfringens D str. JGS1721)	1E-121	99.5% (222/222 aa)	Cytoplasmic Membrane (N)	
CP4_0465	497	CotH protein	CotH protein (Clostridium perfringens D str. JGS1721)	0	99.8% (497/497 aa)	Unknown (N)	Spore coat protein CotH
CP4_0466	685	conserved hypothetical protein	conserved hypothetical protein (Clostridium perfringens D str. JGS1721)	0	98.5% (685/685 aa)	Unknown (N)	
CP4_0467	377	putative heat repeat	heat repeat domain protein (Clostridium perfringens D str. JGS1721)	0	99.5% (377/377 aa)	Unknown (N)	Armadillo-type fold;Carbamoyl phosphate synthetase, large subunit, ATP-binding;HEAT
CP4_0468	466	Putative glycosyl transferase	chitin synthase (Clostridium perfringens D str. JGS1721)	0	99.1% (466/466 aa)	Cytoplasmic Membrane (N)	Glycosyl transferase, family 2

See table 3 for descriptions.

Third, a conserved 5.6 kb NE-specific locus (Table 5; NELoc-3) was identified that harboured five genes, three of which were either absent or significantly divergent from equivalent regions in the reference genomes (Figure 1C). This locus was found on a single contig ranging from 7.4 kb–9.9 kb in four of the NE strains, and on multiple contigs separated by small gaps, based on the CP4 sequence, in the remaining strains.

**Table 5:** Summary of predicted genes in NE pathogenicity locus 3.

Locus_tag^1^	Length (aa)	Predicted Product	Hit Description	E-value	% Identity^2^	Subcellular localization	Conserved domains
CP4_3568	308	hypothetical protein	hypothetical protein A1Q_3418 (Vibrio harveyi HY01)	1E-47	42.2% (282/315 aa)	Unknown (Y)	Chordopoxvirus G2
CP4_3569	213	resolvase/recombinase	resolvase/recombinase (Clostridium perfringens E str. JGS1987)	1E-105	94.7% (207/207 aa)	Unknown (N)	Recombinase, conserved site; Resolvase, N-terminal
CP4_3570	103	conserved hypothetical protein	conserved hypothetical protein (Clostridium perfringens E str. JGS1987)	4E-40	80.6% (103/103 aa)	Unknown (N)	
CP4_3571*	318	bacterial extracellular solute-binding protein	bacterial extracellular solute-binding protein (Clostridium perfringens B str. ATCC 3626)	1E-147	78.8% (316/477 aa)	Unknown (N)	
CP4_3572*	262	NADP-dependent 7-alpha-hydroxysteroid dehydrogenase	NADP-dependent 7-alpha-hydroxysteroid dehydrogenase (Clostridium difficile 630)	5E-94	64.3% (258/262 aa)	Cytoplasmic (N)	Short-chain dehydrogenase/reductase SDR;Glucose/ribitol dehydrogenase;NAD(P)-binding domain

### Features of NE-associated loci

Sequence analyses of the genes in the three loci were performed using BLAST, the Conserved Domains Database (CDD), [28], SignalP [29], pSortB [30], and InterProScan [31] (Tables 3–5). As noted, NELoc-1 is the largest locus of the three, consisting of 37 predicted genes (CP4_3442–CP4_3478); four of which (CP4_3447, 3448, 3460 and 3476) had predicted proteins <100 amino acids (aa) and no significant BLAST hits in GenBank;these were thus excluded from further analyses.

NELoc-1 bears several hallmarks of a horizontally-acquired element. Specifically, we identified two degenerate transposases: one truncated ORF (CP4_3451) with 59% identity to the Tn*1546* transposase [32] and another two ORFs (CP4_3464 and CP4_3465) that, when joined, produce a contiguous alignment (85% identity) with a resolvase/recombinase. CP4_3452 and CP4_3453 also appeared to have been originally joined into a single ORF. The predicted full-length protein encoded by these two ORFs shared 92%–95% identity with a putative transposase/invertase-encoding gene found in the conjugative plasmids pCW3, pCPF4969, pCPF8533etx and pCPF5603. Furthermore, CP4_3477 encoded an apparently truncated swim zinc finger domain protein also present in plasmids pCP8533etx and pCPF5603. Taken together, the presence of these sequences suggested that this locus might be associated with a transposable element and/or plasmid.

Fifteen of the NELoc-1 genes were predicted by pSortb and SignalP to encode cell-surface associated or extracellular proteins, indicating that nearly half of the predicted protein complement of this locus may be exposed to the extracellular environment. Three genes found clustered together near the 5′ end of NELoc-1 (CP4_3471, CP4_3472 and CP4_3473) encode predicted proteins with 66%–76% identity to putative cell surface bound proteins present in several of the *C. perfringens* draft genomes, and/or contain a Cna-like B-region domain. This domain is found in the *Staphylococcus aureus* collagen-binding protein Cna and serves to present the collagen-binding domain away from the cell surface [33]. Located immediately downstream from this cluster is a gene (CP4_3470) encoding a putative sortase, an enzyme responsible for the covalent anchoring of surface proteins to the peptidoglycan layer [34]. Bordering these four predicted surface-associated proteins are two genes (CP4_3478 and CP4_3469) that constitute a putative novel cyclic dimeric guanosine-monophosphate (c-di-GMP) signalling system [35]. Specifically, CP4_3478 encodes a predicted protein containing both GGDEF and EAL domains, responsible for the catalytic activity of diguanylate cyclase (DGC) and phosphodiesterase (PDE) enzymes, respectively [36], while CP4_3469 encodes a protein containing the GGDEF domain only. This system regulates virulence and adhesion in several bacterial pathogens, including *Vibrio cholerae*, *Pseudomonas aeruginosa* and *Staphylococcus aureus* by modulating intracellular levels of the c-di-GMP second messenger molecule. [35], [37].

Just 507 bp downstream from CP4_3469 is a gene that is closely related (64% identity over 1521 aa) to the hypothetical protein CPE1281 of *C. perfringens* strain 13, and more distantly to the discoidin domain protein CPF_1073 (44% identity over 1413 aa) of *C. perfringens* strain ATCC13124. Kulkarni et al. [19] identified a CPE1281 homolog that was recognized by serum from immune broiler chickens. Further analysis of the original protein ID data (obtained from Kulkarni et al) showed that four of the peptides were identical to the predicted protein from NELoc-1 and divergent from the other two chromosomally-encoded homologs. Only one peptide (ITDQNIWENNTYPK) was identical to CPE1281, and this sequence was also shared by the NE homolog (Figure S2).

Of particular interest in NELoc-1 were the two putative leukocidins situated near the middle of the locus (CP4_3459, CP4_3458), which shared 44% and 50% amino acid identity with NetB, as well as similarity with other beta-channel pore-forming toxins that carry the leukocidin domain, including the *C. perfringens* delta-toxin, *C. botulinum* alpha-hemolysin, and the *S. aureus* HlyA and bi-component leukocidins [38], [39]. However, the CP4_3459 and CP4_3458 predicted proteins were only 96 and 114 residues, respectively, while the size of the related pore-forming toxins ranged from 271 (*S. aureus* PVL toxin) to 322 (NetB) amino acids. Altering a single nucleotide to eliminate the intervening stop codon between the two ORFs resulted in a single ORF encoding a predicted 253 amino acid protein which then yielded a contiguous, nearly full-length alignment with NetB. Thus, these two ORFs likely originated from a single leukocidin gene that subsequently acquired a nonsense mutation.

**Figure 2:**
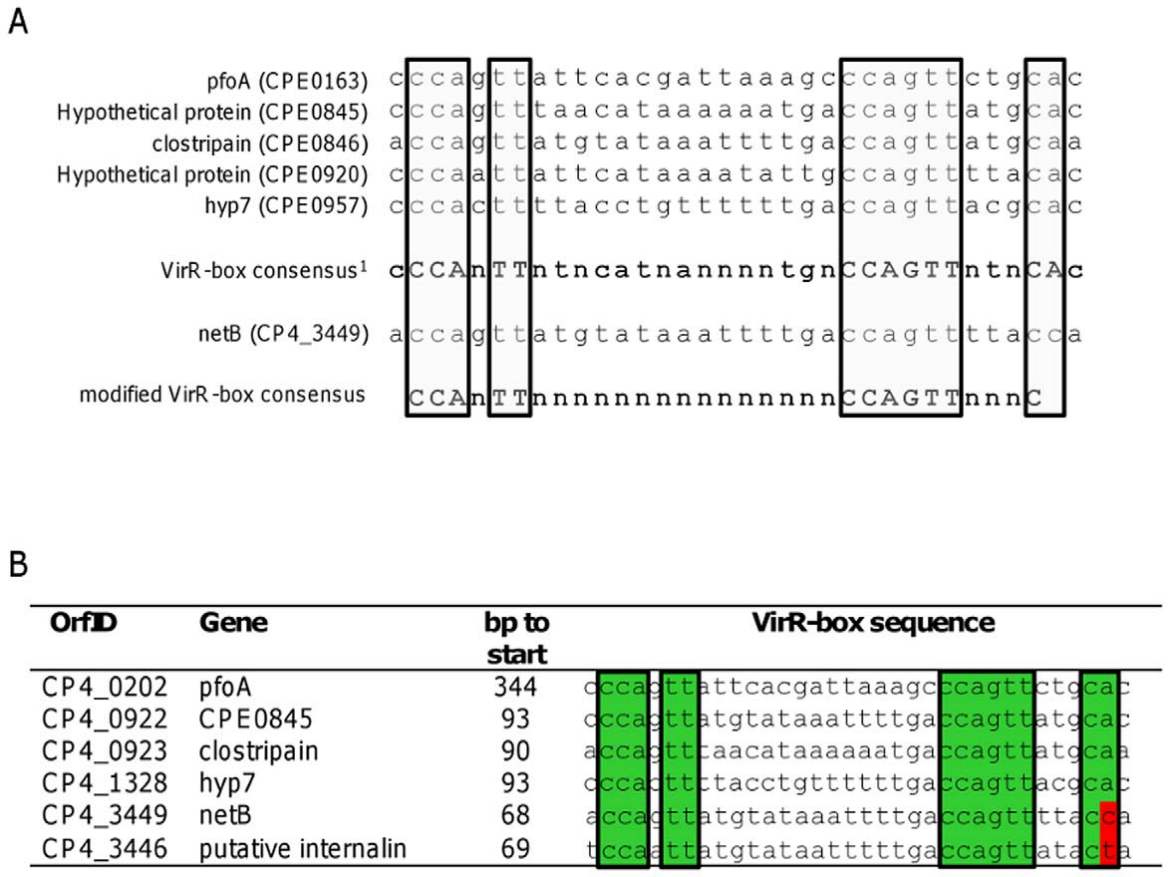
Identification of VirR boxes in NE strain genomes. (A). A modified VirR-box consensus sequence was produced based on the alignment of known VirR-boxes with the VirR-box upstream from *netB*. The VirR-box consensus previously reported by Cheung et al. (2004) [79] is shown in bold, and absolutely conserved nucleotides are in boxes. (B) VirR-boxes identified in CP4, including one novel element upstream of the internalin-like protein gene. Nucleotides that conform with the previously reported VirR-box consensus are in green, and those that do not conform in red.

In addition to these putative toxins, a ricin-domain containing protein (CP4_3450) and an internalin-like protein (CP4_3446) were located near the 3′ end of NELoc-1, flanking *netB* (CP4_3449). It was recently reported that *netB* contains an upstream VirR-box regulatory sequence, and is under direct control of the VirR response regulator [40]. The VirR/VirS two-component signal transduction system regulates several virulence genes in *C. perfringens*, including alpha-toxin, perfringolysin O and alpha-clostripain [41]. By integrating the new sequence information from the element upstream from *netB*, a modified VirR-binding consensus sequence was designed and used to identify additional putative VirR/VirS-regulated genes in the CP4 genome (Figure 2A). One novel putative VirR-box was found 67–69 bp upstream from the putative internalin in all seven NE strains, suggesting that this gene is co-regulated with *netB* by the VirR/VirS system (Figure 2B).

Downstream of the two putative leukocidins (CP4_3459, CP4_3458) were two putative chitinases, which catalyze the breakdown of chitin, a long-chain homopolymer of N-acetylglucosamine. Finally, four additional genes were found at the 3′ end of the locus: two putative transcriptional regulators (CP4_3444 and CP4_3442), a radical SAM-domain protein (CP4_3445), and a metallo-beta-lactamase domain protein (CP4_3443). The metallo-beta-lactamase domain-containing protein superfamily contains ∼6000 members with diverse enzymatic functions, including glyoxylases, flavoproteins, and arylsulfatases, in addition to the beta-lactamases [42]. Antibiotic susceptibility tests revealed sensitivity to beta-lactam antibiotics (Table 1), suggesting the protein encoded by this gene does not possess beta-lactamase activity.

Most genes identified in NELoc-2 are predicted to encode membrane or cell wall associated proteins. One gene cluster of interest was the predicted VTC domain – tubulin – resolvase – VTC domain – tubulin sequence (CP4_0460–CP4_0464). BLAST analyses of the regions flanking the resolvase failed to reveal a repeat region between the two genes that would have been essential for transposition. Also of particular interest was the gene encoding spore coat protein CotH, which may serve as a general marker for *C. perfringens* enteritis strains, since it is also found in the ovine enteritis strain JGS1721. A chitin synthase-encoding ORF may functionally complement two chitinase-encoding ORFs found in NELoc-1; however its relevance is unclear since information about chitin synthases is limited to yeast and higher eukaryotes, where the balance between synthesis and degradation of chitin is essential for cytokinesis [43].

BLAST analyses of NELoc-2 flanking regions indicated this locus is chromosomally located, and identified a 1374 bp region corresponding to genome coordinates 447880–449255 of *C. perfringens* strain ATCC 13124 that had been replaced with the 11.2 kb sequence of NELoc-2. Strain 13 and SM101 BLAST results revealed the replacement of smaller regions of 165 bp and 168 bp, respectively, at the same location in the genome. The presence of a resolvase gene (CP4_0462) in the centre of this locus suggested that it may have originated from a mobile element.

Analyses of NELoc-3 revealed a putative *hdhA* gene (CP4_3572) that encodes 7-alpha-hydroxysteroid dehydrogenase (7-HSDH), a bile-acid inducible enzyme that converts the primary bile acid, chenodeoxycholic acid, into a secondary potentially toxic bile acid, 7-keto-lithocholic acid. A homolog has also been found in *Clostridium difficile*, and its presence may confer a survival advantage through the suppression of commensal bacteria by the HSDH-mediated production of a toxic metabolite [44]. Another gene of interest was that predicted to encode an extracellular solute binding protein (ESB) (CP4_3571). The predicted protein was found to be 100% conserved among NE strains, compared to a maximum of 78% identity to the *C. perfringens* strain ATCC 13124.

The predicted protein encoded by CP4_3570 shared significant similarity with hypothetical proteins pCW3_0046 (79% identity) and pCPF5603_58 (76% identity) encoded by *C. perfringens* plasmids pCW3 and pCPF5603, respectively. This finding, along with the identification of a putative resolvase (CP4_3569) in this locus, suggested NELoc-3 may have arisen from a transposable element and is plasmid-encoded. Unlike NELoc-2, we could not identify flanking genomic sequences on any contigs carrying NELoc-1 or -3, and neither locus aligned with the optical map generated for the CP4 chromosome.

### Characterization of large plasmids of NE strains

The identification of plasmid sequences in NELoc-1 and -3 prompted efforts to identify and characterize the plasmids of our NE strains. A number of *C. perfringens* toxin genes are associated with large plasmids, including beta-toxin (*cpb*), epsilon toxin (*etx*), beta2-toxin (*cpb2*), iota toxin (*iap/ibp*) and, variably, *C. perfringens* enterotoxin (*cpe*) [14], [15], [45]–[47]. Recent characterization of toxin plasmids of *C. perfringens* type A, B, D and E strains [13]–[16] have revealed a toxin plasmid family, designated the pCPF5603-like toxin plasmids [15], that share a similar structure; this consists of a conserved backbone region of ∼30 kb carrying the transfer of clostridial plasmid (*tcp*) locus for conjugative transfer, and a ∼20–40 kb variable region that encodes toxins [13], [15], [48]. The complete sequences of three members of this family (pCPF5603, pCPF4969, pCP8533etx) have been determined [13], [15]. In addition, the tetracycline resistance plasmid, pCW3, is closely related to this plasmid family, sharing the conserved core but carrying a resistance locus in place of the toxin-encoding loci [13], [15], [48].

**Figure 3:**
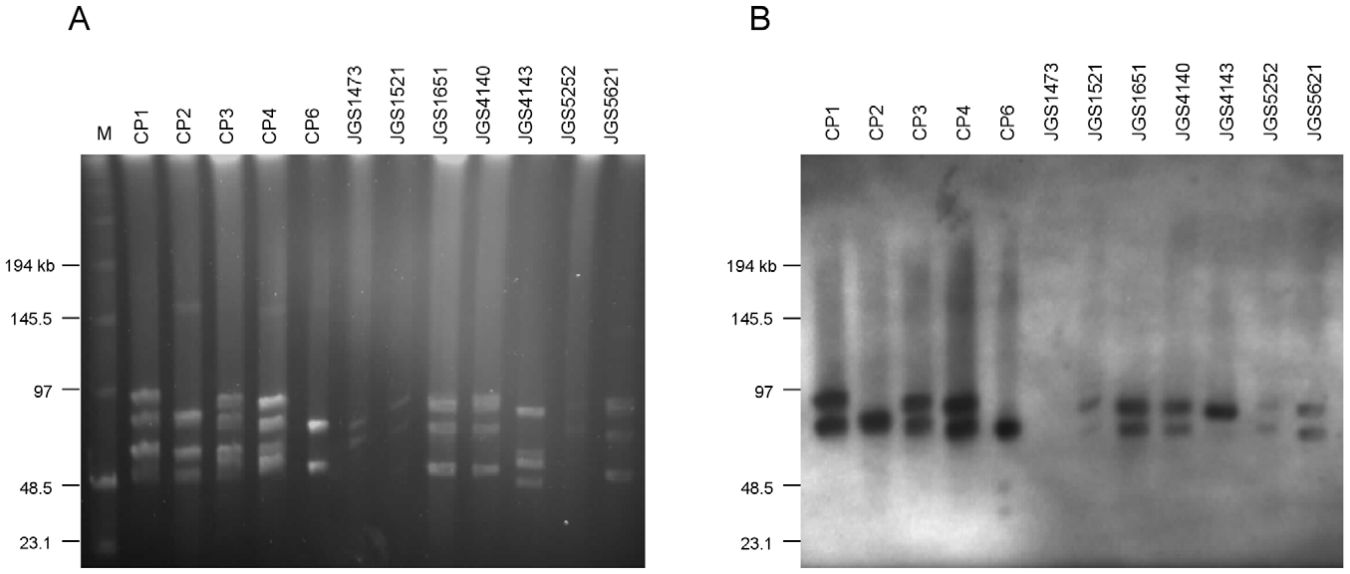
PFGE and Southern blot analyses of plasmids from *C. perfringens* poultry strains. (A). Agarose plugs containing DNA isolated from the eight sequenced poultry strains were digested with NotI and subjected to PFGE. (B). Southern blotting was performed with DIG-labelled probes for *netB* and *hdhA*. Results from both *netB and hdhA* probes are shown overlayed. In all lanes with two bands, the upper band represents *netB* and the lower band *hdhA*. Both probes hybridized to the same band in CP2. In CP6, only the *hdhA* probe hybridized, while in JGS4143, only the *netB* probe hybridized.

To first determine if our NE strains carried large plasmids, DNA from our eight sequenced poultry isolates, as well as three additional virulent NE isolates (CP1–3) and one avirulent NE isolate (CP6), were subjected to PFGE. *In silico* restriction endonuclease analysis of the genomes of SM101 and ATCC13124, as well as pCPF5603, pCPF4969 and pCP8533etx, revealed that *Not*I cleaved the genomes at no more than one location, whereas the plasmids were cleaved exactly once; this restriction enzyme was therefore chosen to linearize the plasmids prior to PFGE. The PFGE profiles of the virulent NE type A strains digested with *Not*I revealed two to five large plasmids ranging in size from ∼45 kb–90 kb in all strains (Figure 3A). Only one NE strain, JGS5252, carried just two large plasmids, as did the healthy chicken isolate, JGS1473, and the avirulent NE isolate, CP6. Three of the NE strains (CP2, CP4 and JGS5621) also carried a larger plasmid of ∼130 kb–150 kb. Further PFGE experiments using undigested DNA, which allows plasmid but not chromosomal DNA to enter a pulsed field gel [15], confirmed the PFGE results (data not shown).

To determine if the identified plasmids were related to the pCPF5603-like plasmids of *C. perfringens*, digoxygenin (DIG) -labelled probes for three genes (*cna*, *dcm*, *tcpF*) conserved among all sequenced plasmids of this family were used in Southern blotting experiments of the pulsed-field separated plasmids from five NE strains (CP1–4, CP6). We observed that when *Not*I-digested genomic DNA was probed, all bands, with the exception of the larger ∼150-kb bands, hybridized specifically with one or more of the *cna-*, *dcm-*, or *tcpF-*specific probes, confirming the plasmid identity of these PFGE bands (data not shown). In particular, the *tcpF*-probe, which is specific for the *tcp* conjugation locus, hybridized to all the ∼45 kb–90 kb plasmids in each strain examined, indicating that they carry this locus and may therefore be conjugative.

Surprisingly, many genes that make up the conserved region in this plasmid family, including constituents of the *tcp* locus, could not be found in the CP4 draft genome. Repetitive sequences can confound assembly algorithms, so we reasoned that these sequences might be misassembled if they were harboured on multiple plasmids in a single *C. perfringens* strain. Mapping of CP4 Solexa reads to the three sequenced plasmids using the reference assembler, Maq (http://maq.sourceforge.net), revealed that CP4 does in fact carry sequences corresponding to all of the conserved regions, including the *tcp* locus (Figure S3). This provides further evidence that the plasmids carried by our NE strains are closely related to the previously characterized *C. perfringens* toxin-encoding plasmids.

### Plasmid localization of NELoc-1 and 3

To test the hypothesis that NELoc-1 and -3 were located on one of the large plasmids identified in the NE strains, DIG-labelled PCR probes based on *netB* (NELoc-1) and *hdhA* (NELoc-3) were used in Southern blotting experiments to probe membranes of transferred PFGE-separated plasmids from all 12 poultry strains. Hybridization of the *netB*-probe to a single ∼80 kb–90 kb plasmid was observed in all NE strains, but not in the avirulent strains CP6 and JGS1473 (Figure 3B). The *hdhA* probe hybridized to a different plasmid of ∼70 kb–80 kb in nine of the ten virulent NE strains, as well as CP6, but not in the healthy chicken isolate JGS1473. Five of the strains (CP1–4, CP6) were also examined in Southern blotting experiments with a probe based on *cpb2*, and an identical hybridization pattern to that of the *hdhA* probe was observed in these strains (data not shown). Interestingly, the *netB*, *hdhA* and *cpb2* probes co-hybridized to the same ∼80 kb band in strain CP2, suggesting that NELoc-1 and -3 may either be on the same plasmid in this strain, or alternatively, on separate but similarly sized plasmids that are not resolvable under the PFGE conditions used here. Thus, NELoc-1 and -3 are harboured on separate large plasmids in most NE strains, and the latter plasmid also carries *cpb2*.

**Figure 4:**
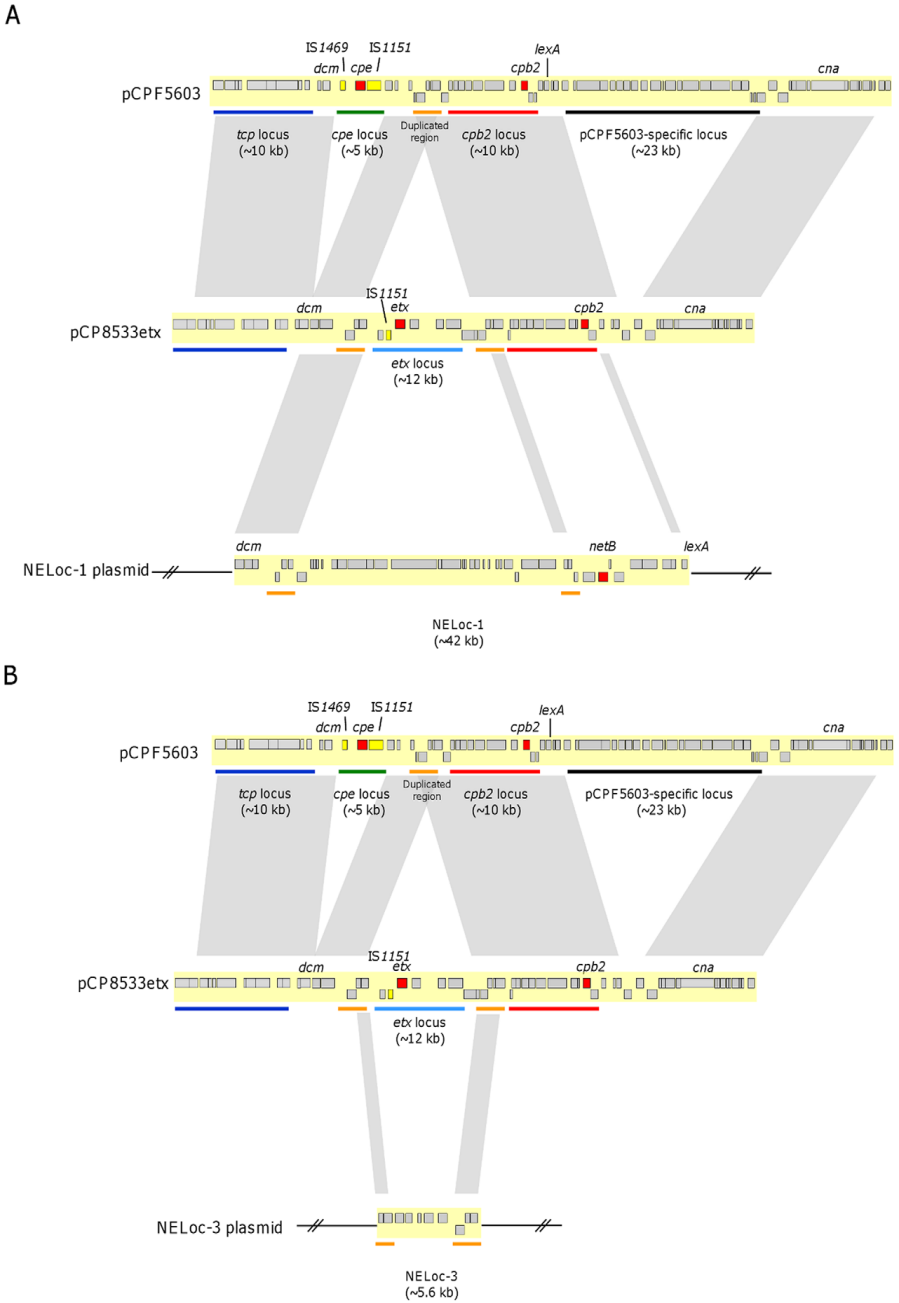
Plasmid location of NELoc-1 and -3. Nucleotide sequence alignments of pCPF5603, pCP8533etx and (A) NELoc-1 or (B) NELoc-3 were generated with Artemis Comparison Tool (ACT). The small boxes represent ORFs, with toxin genes colored in red and IS*1151* or IS*1469* genes in yellow. Grey boxes connecting the different sequences represent regions with sequence similarity. Colored bars underneath each sequence represent different loci as follows: blue, *tcp*; green, *cpe*; red, *cpb2*; black, pCPF5603-specific; light blue, *etx*. Orange bars represent the sequence found duplicated in pCP8533etx and NELoc-1 and -3 sequences.

We next sought to determine the insertion sites of NELoc-1 and -3 within their respective plasmids. Outward-facing PCR primers were designed at both ends of NELoc-1 based on the CP4 sequence, which were used in combination with primers designed from various genes common to pCPF5603 and pCPF4969 to amplify flanking junction regions via long-range PCR. A 9 kb product was amplified using primers sigP-F and dcm-F, linking the signal peptidase I (CP4_3474) with *dcm*. At the opposite end of NELoc-1, use of primer pair blac-F and lexA-F amplified a 2.7 kb fragment, thereby linking this end to the *lexA* repressor gene. These PCR products were subsequently sequenced and used to extend the CP4 NELoc-1 sequence at both ends. Alignment of this extended locus to the three sequenced pCPF5603-like plasmids revealed a structure and insertion site most similar to the variable region of pCP8533etx, which carries both a *cpb2* and *etx* locus (Figure 4A). In pCP8533etx, the *etx* locus can be delineated from the rest of the variable region by three genes that are directly repeated on either end; these genes are present as single copies in pCPF5603, pCPF4969, and pCW3. A similar duplication is observed in NELoc-1, effectively delimiting this locus into one ∼30 kb unit corresponding to the *etx* locus, extending from the DGC to chitinase B- encoding genes, and another ∼12 kb unit corresponding to the *cpb2* locus, extending from the Tn*1546*-like transposase to the MarR transcriptional regulator-encoding genes, and including *netB*.

For NELoc-3, sufficient flanking plasmid sequence was present in the contig from JGS4140 to define its putative insertion site (Figure 4B). This insertion site also corresponded to that of the *etx* locus of pCP8533etx and was bordered by the same direct repeats described above. Notably, neither locus contained IS*1151* or IS*1470*-related sequences, in contrast to previously described loci containing *cpe*, *etx*, *iap/ibp* and *cpb* [14], [15], [17], [49].

### Diversity analysis of the three NE loci among poultry strains

Multiple alignments of each locus from our seven sequenced NE strains revealed a striking level of conservation among these sequences between the different strains (Figure S4). To exclude the possibility that this conservation was due to a clonal relationship, PFGE profiles of genomic DNA were determined for the eight sequenced strains (Figure S5A). In addition, a phylogenetic tree based on the alignment of the total predicted proteome sequence of each strain was constructed via CVtree [50] (Figure S5B). Three of the strains (JGS4140, JGS1651 and JGS5621) were found to be closely related by both methods, but none appeared to be clonally related and the remaining five strains were significantly divergent.

In most of the sequenced strains, the three NE loci were fragmented into multiple contigs and it was therefore not possible to assess their uniformity based on sequence data alone. An overlapping PCR assay was therefore developed [46] to assess the diversity of the three loci in our sequenced strains and the four additional poultry isolates, and at the same time confirm the sites of insertion. A set of fifteen PCR reactions were developed for NELoc-1, five reactions for NELoc-2 and four reactions for NELoc-3, all based upon CP4 and JGS4140 sequences and spanning each entire locus. The reactions at either end of each locus were designed to extend into the flanking sequences to verify the site of insertion (Figures S6, S7, and S8).

PCR reactions for NELoc-1 amplified most of the expected products from the ten virulent NE strains (Figure S6), indicating near-uniformity of this locus across these strains. In CP1, reaction NEL1-6, encompassing leukocidin I and II, was ∼1.2kb larger than expected, and sequencing of this amplicon revealed an additional ∼1kb ORF encoding an intact ISCpe7 transposase upstream from leukocidin I. Four of the reactions for CP2 were negative, indicating that the region encompassing the chitinase and leukocidin genes was absent in this strain. Two of the reactions from CP6, spanning the chitinase and leukocidin genes and one reaction from JGS1473, encompassing the CPE1281 homolog, amplified the expected product. The latter result was consistent with the findings from the comparative genome analyses, which identified a region from NELoc-1 to be partially intact in JGS1473, consisting of the sortase and cell-wall surface protein-encoding genes, in addition to the CPE1281 homolog. This suggests that portions of NELoc-1 may be present in these avirulent strains, possibly on a modified *netB* plasmid, or chromosomally located.

The PCR products generated from the NELoc-2 and -3 assays were identical for all of the NE strains, as well as the avirulent strain CP6, and consistent with the predicted sizes (Figure S7, S8).

## Discussion

The pathogenesis of poultry NE has been the subject of considerable investigation in recent years, following reassessment of the role of CPA and subsequent identification of the novel pore-forming toxin NetB [3], [7]. We show here for the first time that *netB* resides on a ∼42 kb plasmid-encoded pathogenicity locus (NELoc-1) harbored specifically by NE strains. In addition, we identified two other loci associated with NE, NELoc-2 and -3, the latter of which also resides on a plasmid that is similar to, but distinct from, that carrying *netB*. Sequence alignments of NELoc-1 to -3 from seven strains revealed their striking uniformity among diverse NE isolates (Figure S4). The high level of sequence conservation was surprising and suggests that these plasmids arose from a recent evolutionary event. Southern blotting results with a *tcpF* probe, together with the detection of the *tcp* locus sequence in the CP4 Solexa reads, indicate that the plasmids carrying NELoc-1 and -3 also carry this conjugation locus. The observation that these unrelated NE strains carry nearly identical plasmid loci is consistent with the hypothesis that the plasmids were acquired through conjugative transfer. This study provides further evidence of the remarkable contribution of closely related multiple plasmids to the infection biology of *C. perfringens* in its ability to cause distinct and serious enteric infections in different animal species [13]–[15], [17]

While functional studies are ultimately required to elucidate the contribution of these genes to NE pathogenesis, sequence analysis can assist greatly in this effort. NELoc-1 encodes 37 putative proteins, of which 25 have no apparent counterpart in any of the sequenced *C. perfringens* strains. An internalin-like protein is located immediately upstream from *netB* containing a leucine-rich repeat (LRR) domain (CP4_3446) with 30–35% identity to Internalin F, D and A of *Listeria monocytogenes* and putative internalins of *Bacillus* spp.. Originally identified in *L. monocytogenes* as surface-anchored proteins required for attachment and invasion [51], internalins now constitute a multi-gene family, with 25 members identified in the *L. monocytogenes* genome alone [52]. Genome sequencing projects have also revealed internalin-like proteins in *C. botulinum*, *C. perfringens and C. tetani*, though none have been functionally characterized [53], [54]. The hallmark LRR domain, responsible for the horseshoe-like conformation of the protein, is found in a functionally diverse set of proteins, and is typically involved in protein-protein interactions [55]. The presence of a VirR-box upstream of the gene suggests that it may be co-regulated with *netB*, and therefore also involved in NE pathogenesis.

Immediately downstream from *netB* is a gene encoding a 391 aa protein (CP4_3450) containing a beta-trefoil domain similar to that of the carbohydrate-binding ricin B subunit [56]. BLAST searches revealed similarity to the ricin-like domains of a diverse set of proteins, including pierisin from *Pieris rapae* (27% identity over 334 aa) and MTX from *Bacillus sphaericus* (30% over 286 aa), which are related ADP-ribosylating toxins [57], the Cyt1Ca insecticidal pore-forming toxin from *Bacillus thuringiensis* (28% over 264 aa) [58], and the HA-1 non-toxin hemagglutinin of *C. botulinum* (33% identity over 279 aa) [59], [60]. These proteins share a similar secondary structure consisting of the C-terminal ricin-like domain responsible for carbohydrate binding and, with the exception of HA1, a distinct N-terminal active-site domain. The ∼130 aa N-terminal region predicted in CP4_3450 did not share sequence similarity with any protein found in GenBank, and a putative function could therefore not be assigned.

CP4_3446, *netB* and CP4_3450 are oriented divergently from the majority of the other genes in NELoc-1. Given this common orientation and evidence for co-regulation, it is plausible that these three genes are co-transcribed, and may act in unison. One possible scenario is that CP4_3446 and CP4_3450 act directly with NetB to enhance its pore-forming activity, although this seems unlikely as the cytotoxicity of purified recombinant NetB was found to be comparable to that of the native protein [7]. Alternatively, they may instead be involved in recognition of eukaryotic cell surface receptors, as suggested by the presence of carbohydrate- (CP4_3450) and protein- (CP4_3446) binding domains and similarity to proteins known to function in this capacity.

The presence of two putative chitinases (CP4_3454 and CP4_3455) is intriguing and suggests a possible role for chitin hydrolysis in NE pathogenesis. This abundant polysaccharide is an insoluble homopolymer of N-acetylglucosamine (GlcNac) found in the exoskeletons of invertebrates and the cell walls of fungi. Chitinases have been detected in other clostridial species, including *C. paraputrificum* [61], [62] and *C. botulinum* [54], both of which have chitinolytic activity, but to our knowledge this is the first example of a chitinase gene identified in *C. perfringens*. These enzymes are typically produced by bacteria that use chitin as a carbon source, or are pathogens of insects or fungi [63]. The incorporation of chitin in the diets of chickens has been shown in some cases to improve growth performance [64], [65]. The putative chitin synthase (CP4_0468) in NELoc-3 may work in conjunction with the two putative chitinases (CP4_3454 and CP4_3455) in NELoc-1 to take advantage of the chitin as an extra carbon source. The degradation of chitin into GlcNac may also confer a survival advantage to the NE strains since it inhibits the adhesion of probiotic lactobacilli to chicken intestinal mucus [66]. Chitin synthase may be needed to convert excess GlcNac back into chitin if the need arises. Intriguingly, in the intestinal bacterial pathogen *V. cholerae* the product of a putative chitinase gene with GlcNAc-binding activity has been shown to act as a common adhesion molecule for both chitinous and intestinal surfaces [67]. GlcNAc forms the bulk of the carbohydrates present in intestinal mucin and this protein has been shown not only to be involved in intestinal colonization in mice but also to increase the quantity of intestinal mucin produced [68], suggesting a possible role for putative chitinases in intestinal colonization in NE.

Also of interest were two putative leukocidins (CP4_3458 and CP4_3459) that exhibited close similarity to NetB, and to a lesser extent to the related *C. perfringens* pore-forming beta and delta toxins [38], hemolysin II of Bacillus *spp.*, and the bi-component leukocidins of *S. aureus*. The two genes are in the same reading frame; elimination of the intervening stop codon forms a single gene with an intact leukocidin domain, and the predicted protein forms a nearly full-length alignment with NetB. These genes may therefore represent an evolutionary relic of a previous *netB* duplication event, or even a now-defunct second member of an original bi-component hetero-oligomeric pore-forming toxin.

The discovery of a potential novel c-di-GMP signaling system, composed of putative PDE (CP4_3469) and DGC (CP4_3478) genes, as well as several nearby genes for putative cell surface proteins (CP4_3471, CP4_3472 and CP4_3473) was of particular interest. This signaling system has recently been shown to play a central role in governing adhesion, motility, and virulence in pathogenic bacteria such as *P. aeruginosa*, *Salmonella* Typhimurium, *S. aureus* and *V. cholerae* [35], [37], [69], [70]. The c-di-GMP nucleotide acts as a second messenger by binding and regulating downstream effectors that are only now being elucidated [71]. Intracellular c-di-GMP levels are modulated through the opposing activities of a DGC and PDE, responsible for its synthesis and degradation, respectively [36]. Both CP4_3469 and CP4_3478 contain the GGDEF domain (responsible for DGC activity), while CP4_3469 also contains an EAL domain (responsible for PDE activity). It is therefore likely that CP4_3478 functions as a DGC, while CP4_3469 may have both PDE and DGC activities, although it is common for PDEs to also carry a non-functional GGDEF domain [34]. CP4_3478 also contains a signal peptide and several N-terminal transmembrane regions, making it a good candidate for an integral membrane DGC. The cluster of four surface-associated protein genes found between CP4_3469 and CP4_3478 may encode adhesins under the control of this system. Two of the genes were also found in avirulent strain JGS1473, however, suggesting a limited role in virulence. It is well-recognized that large numbers of *C. perfringens* will coat the damaged intestinal epithelial surface of chickens with NE [72], but little to no information exists on the specificity of this adherence.

The recognition of a homolog of the large hypothetical protein CPE1281 from Strain 13 was among many of the unexpected discoveries reported here. Earlier work based on mass spectrometry of proteins expressed by virulent but not avirulent *C. perfringens* recovered from birds with NE [19] mis-identified the protein as CPE1281, instead of the NE-specific homolog. Immunization of chickens with the most immunogenic part of the CE1281 protein gave excellent protection of birds against experimentally-induced NE [4] Epitope-mapping of the CPE1281 protein (not the NE 1281 homolog described here) identified immunodominant regions of the CPE1281 protein, which include the possible zinc-binding signature region of this hypothetical protease in both proteins (G—HELGHNF), which were used in immunization when expressed from a *Salmonella* vaccine vector [20]. Further work is required to determine whether the efficacy of this protein in protecting birds against NE is the result of cross-protection against the NE CPE1281 homologue.

Two homologs of tubulin/FtsZ (CP4_0461 and CP4_0464) are found in NELoc-2 sharing 33% and 36% protein identity, respectively, with tubulin/FtsZ common to other *C. perfringens* strains. The homologs might be similar in function to the *Bacillus thuringiensis* tubulin homolog TubZ, which is proposed to facilitate plasmid segregation by coupling with plasmid DNA, via DNA-binding proteins, and causing plasmids separation [73]. In this case, NELoc-2 may not be specifically involved in virulence, but might instead contribute to the preservation of the NELoc-1 and -3 virulence plasmids via the two tubulin/FtsZ homologs. This may help explain the frequency of plasmid occurrence in NE stains from different sources.

Extracellular solute-binding proteins (ESBs) of bacteria may serve as chemoreceptors, recognition constituents of transport systems, and initiators of signal transduction pathways, suggesting several possible roles for the conserved ESB gene in NELoc-3 [74]. The NADP-dependent 7-alpha-hydroxysteroid dehydrogenase found in NELoc-3 shares 64.3% protein identity with the 7-HSDH of *C. difficile*. It may similarly be responsible for converting the primary bile acid, chenodeoxycholic acid, into the secondary bile acid, 7-keto-lithocholic acid, which is potentially toxic to other members of the microbiota [44]., though it is not clear how NE strains are protected.

PFGE analyses revealed the presence of two to five plasmids, ranging in size from ∼45 to 150 kb, in ten NE isolates of known virulence. Southern blotting of pulsed-field gels revealed *netB* (NELoc-1) on a ∼80 kb–90 kb plasmid in virulent isolates and *hdhA* (NELoc-3) on a separate ∼70 kb–80 kb plasmid. Only in CP2 were *netB* and *hdhA* apparently co-localized to the same plasmid. Almost all *C. perfringens* toxins are now known to be transcribed from genes on large plasmids. These plasmids share a conserved backbone carrying a *tcp* conjugation locus, and it has been suggested that they be referred to as the pCPF5603-like toxin plasmids [15]. Alignment of NELoc-1 and -3 and flanking sequences with members of this family reveal a shared structure and site of insertion, most closely resembling pCP8533etx, suggesting that the NELoc-1 and -3 plasmids are genuine members of this growing plasmid family. Toxin genes present in other pCPF5603-like toxin plasmids, including *cpe* [13], *etx* [15] and *iap/ibp* [14], are in close proximity to IS*1151* sequences, and the detection of circular transposition intermediates carrying both IS*1151* and toxin gene sequences [14], [16], [17] has led to the suggestion that these elements mobilize many of the *C. perfringens* toxin genes [15]. Contrary to this, related sequences were not found near either NELoc-1 or NELoc-3, which may indicate that these loci were acquired through a different mechanism, possibly involving a distinct transposable element.

Interestingly, the plasmid on which NELoc-3 resided also contained *cpb2*, which has been previously identified in NE isolates [18]. The role of the Cpb2 toxin in enteric disease including NE is unclear. Since *cpb2* is widespread in *C. perfringens*, the sequencing approach taken here failed to identify this gene as part of the “NE signature”, but does not preclude the involvement of it, or other toxins such as CPA, in NE.

Overlapping PCR studies of NELoc-1, -2 and -3 demonstrated a conserved organization and site of insertion among 10 type A NE strains (Figures S6, S7, and S8). Only CP2 had a different profile, with the absence of ∼6kb encompassing the chitinase B and leukocidin I and II genes. This may be indicative of the stepwise evolution of the virulence loci of *C. perfringens* plasmids through addition or deletion of specific segments bordered by IS elements.

In contrast to NELoc-1, both NELoc-2 and -3 were also found in the avirulent strain CP6. Interestingly, this strain was originally recovered from a chicken with NE, but was subsequently avirulent in experimental challenge studies [18]. The presence of intact NELoc-2 and -3 in this strain suggests that it may have lost at least part of NELoc-1; anecdotally, loss of virulence is not uncommon in NE isolates and the recognition of the plasmid basis of virulence provides a simple explanation for this well-recognized phenomenon. The conservation of the NELoc-3 plasmid in NE isolates suggests that both plasmids contribute to the virulence of NE strains. Further work is required to characterize this plasmid.

In conclusion, this study has contributed significantly to our understanding of the pathogenetic basis of NE in chickens. The majority of virulence-associated genes identified here are carried on different plasmids in virulent NE strains of *C. perfringens*, and loss of the locus encoding *netB* appears to attenuate virulence. The findings of this study also support previous evidence for a common ancestor of the *C. perfringens* toxin plasmids. Functional characterization of putative virulence genes identified here may provide significant insights into the mechanism of NE pathogenesis.

Genes identified as potential virulence factors can be cloned into suitable vectors for expression of recombinant proteins. It may be determined that codon optimization is necessary to improve levels of protein expressed. Following isolation and purification, the proteins could be combined with an appropriate adjuvant, and used to immunize chickens, providing protection against infection caused by *Clostridium perfringens*. Alternatively, they could be cloned into vectors suitable for direct vaccination of birds as nucleic acid vaccines. They could also be cloned into viral vectors, which could be transfected into host cells for the production and purification of recombinant virus, which could then be used to immunize birds. Finally, they could be cloned into bacterial expression systems, which could then be isolated as recombinant bacteria, which could also be used to immunize birds.

## Materials and Methods

### Bacterial strains and genomic DNA isolation

*Clostridium perfringens* strains used in this study are described in Table 1. CP1–4 and CP6 are field isolates from NE cases in Ontario, and were obtained from D.A. Barnum, Department of Pathobiology, University of Guelph. The virulence of strains CP1–4 has been confirmed, while CP6 is known to be avirulent [18]. Genomic DNA was isolated from 5 ml of overnight culture in Brain Heart Infusion (BHI) at 37°C under anaerobic conditions. Bacteria were pelleted and lysed for 5 min in lysis buffer (5mg lysozyme/ml, 50 mM Tris, pH 8, 25% sucrose), treated with RNase A (40 ug/ml) at 37°C for 30 min followed by proteinase K (300 ug/ml) overnight at 37°C. Samples were extracted with equal volumes of phenol, phenol∶chloroform∶isoamyl alcohol (25∶24∶1), and chloroform∶isoamyl alcohol (24∶1), precipitated with isopropanol, and then resuspended in TE buffer. Following a second precipitation with 2 volumes of 95% ethanol, the pellets were washed twice with 70% ethanol and resuspended in water. This crude DNA extract was further purified with the DNeasy kit (Qiagen, Mississauga, Canada) according to the manufacturer's instructions. The quality of the genomic DNA was assessed by agarose gel electrophoresis and the identity confirmed by PCR amplification of *cpa* and *netB*.

### High-throughput sequencing, assembly, and annotation

CP4 was sequenced using the Illumina/Solexa Genome Analyzer System in both single-end and paired-end runs, each generating 34–36-bp reads. Each run was assembled separately with the *de novo* short-read assembler Velvet v0.7.48 [23], using empirically optimized parameters, and the two assemblies subsequently combined using the minimus2 script from AMOS v2.0.8 [75] The contigs were oriented and ordered according to the finished reference genome for ATCC 13124 using Mummer v3.21 [24] and custom perl scripts. Additional joining of contigs was accomplished by searching for short overlapping sequences between neighbouring contigs. In addition, an optical map of CP4 (OpGen, Inc.) was used to identify assembly errors, which were manually corrected where possible. Contigs that did not align to either the reference genome or the optical map were placed at the end of the sequence.

The contigs were joined into a pseudomolecule with the linker sequence NNNNNCACACACTTAATTAATTAAGTGTGTGNNNNN and submitted to the JCVI Annotation Service, where it was run through the prokaryotic annotation pipeline. Included in the pipeline are gene finding with Glimmer, Blast-extend-repraze (BER) searches, HMM searches, TMHMM searches, SignalP predictions, and automatic annotations from AutoAnnotate. Putative domains were predicted with the Conserved Domains Database (CDD) [28] and InterProScan [31]. Manual correction of the annotations was performed for the genes found in the three NE loci.

NE locus regions identified as potentially misassembled or containing sequencing errors were resolved by capillary sequencing of PCR products. Primers used for PCR and sequencing are listed in Table S1.

### Identification of NE-associated genes

Sequences common to NE strains JGS4143 and CP4, but absent from nine reference genomes, were identified via MUMmer v3.21, Bedtools v2.1 [76], and custom perl scripts. Briefly, the draft genome sequence of CP4 was aligned to each reference genome using NUCmer with the parameters -l 10 -c 30 -b 500 -maxgap 1000. The resulting coordinate files were converted to Bed format and alignments <50bp were removed using custom perl scripts. The coordinates were inverted using the complementBed script from the Bedtools v2.1 package, producing CP4 genomic regions that did not align to each reference genome. These were successively intersected using intersectBed to give the regions found only in CP4, and finally intersected with the regions that aligned between CP4 and JGS4143 to output those common to the two NE strains, but not found in any of the reference genomes. Custom perl scripts were used to convert the coordinate files to EMBL format for viewing in Artemis v11 [77], and to extract the genes overlapping these intervals from the annotated genome. To confirm that these genes were unique to the NE strains, the nine reference genomes were searched for potential orthologs using OrthoMCL v2.0 [25], which identifies orthologs based on clustering of reciprocal best BLAST hits, using cut-offs of E-value <1e-5 and percent match >50%.

The three finished genomes were downloaded from the NCBI ftp site (ftp://ftp.ncbi.nih.gov/genomes/Bacteria/), while the contigs for the six draft genomes were downloaded separately from GenBank and combined into a single multi-fasta file.

### Antimicrobial susceptibility testing

*C. perfringens* strains were tested for antimicrobial susceptibility by the agar disc diffusion method or E-test. The following antimicrobial agents (Oxoid, Hampshire, UK) were tested: ampicillin (10 mg), bacitracin (E-test), chloramphenicol (30 mg), clindamycin (2 mg), erythromycin (15 mg), nalidixic acid (30 mg), teicoplanin (30 mg), tetracycline (30 mg), vancomycin (5 mg). MIC values were analysed after incubation at 37°C overnight in anaerobic conditions.

### Pulse Field Gel Electrophoresis (PFGE)

PFGE was performed to analyze genetic diversity and the presence of plasmids in 12 poultry *C perfringens* strains, as described [14]. Briefly, DNA plugs for PFGE were prepared from overnight cultures of *C. perfringens* grown in TGY and the bacterial pellets incorporated into a final agarose concentration of 1% in PFGE-certified agarose (Bio-Rad Laboratories, Hercules, CA). Plugs were incubated overnight with gentle shaking at 37°C in lysis buffer (0.5M EDTA pH8.0, 2.5% of 20% sarkosyl (Fisher Scientific, Fair Lawn, NJ), 0.25% lysozyme (Sigma-Aldrich Co., St. Louis, MO) and subsequently incubated in 2% proteinase K (Roche Applied Science) buffer for 2 days at 55°C. One third of a plug per isolate was equilibrated in 200 µL restriction buffer at room temperature for 20 min and then digested with 10 U of *Not*I (New England Biolabs, Ipswich, MA) at 37°C overnight. Electrophoresis was performed in a 1% PFGE-certified gel and separated with the CHEF-III PFGE system (Bio-Rad Laboratories, Hercules, CA) in 0.5× Tris-borate-EDTA buffer at 14°C at 6 V for 19 h with a ramped pulse time of 1 to 12 s. Gels were stained in ethidium bromide and visualized by UV light. Mid-Range II PFG markers (New England Biolabs) were used as molecular DNA ladder.

### Preparation of DIG probes and Southern blot hybridizations of PFGEs

DNA probes for all Southern blots steps were labelled by PCR amplification in the presence of digoxigenin-11-dUTP (DIG; Roche Applied Science) according to the manufacturer's recommendation. DNA probes were amplified from *C. perfringens* strain CP4. DNA probes for *netB* and *hdhA* genes were prepared with specific internal primers (Table S1). DNA from PFGE gels was transferred to nylon membranes (Hybond-N; Amersham). DNA hybridizations and detection were performed by using the DIG labelling and CSPD substrate according to the manufacturer's recommendation (DIG system user's guide for filter hybridization, Roche). For Southern blot hybridizations, nylon membranes were prehybridized for at least 2 h at 42°C in hybridization solution without labelled probe and then hybridized separately at 42°C with specific DNA probes for 16 h. The membranes were washed at 68°C under high-stringency conditions. For each different DIG- labelled probe, the membrane was first stripped with 0.2 N NaOH and 0.1% sodium dodecyl sulfate, incubated with prehybridization solution, and then reprobed.

### Overlapping PCR analysis of NE locus 1–3

A battery of PCR reactions was performed to assess the conservation of NELoc-1–3 among 12 poultry isolates. For NELoc-1 and 2 reactions, a ready-to-use PCR mixture of Platinum PCR SuperMix high-fidelity kit (Invitrogen, Burlington, ON, Canada) was used in a 25 µl reaction containing 0.8 µM of each primer. A touch-down PCR program was used: 94°C for 3 min, 35 cycles of 94°C for 15 s, 65°C to 50°C for 15 s/cycle (the annealing temperature is decreased by 1°C every cycle until 50°C), extension at 68°C for 5 min, and finally, 68°C for 10 min. For longer range fragments the extension time was increased to 15 min. For the NELoc-3 reactions, each PCR mixture contained approximately 50 ηg of template DNA, 0.5 U of Platinum Taq High Fidelity (Invitrogen), 0.2 mM dNTPs, 1× PCR buffer, 2 mM MgSO_4_, and 0.2 µM of each primer in a 25µl reaction. The reaction mixtures were subjected to the following amplification conditions: one cycle of 95°C for 5 min; 35 cycles of 95°C for 30s, 53°C for 30s, and 68°C for 5 min; followed by one cycle of 72°C for 10 min. All primers used are described in Table S2. PCR product sizes were determined by agarose gel electrophoresis and visualization by ethidium bromide staining and fragments that did not match the expected size were sequenced.

### Mapping of Solexa sequencing reads to plasmid sequences

In order to detect plasmid sequences in the CP4 genome that could not be assembled by Velvet, the Solexa reads were mapped to each of the published *C. perfringens* plasmid sequences using Maq v0.7.1 (http://maq.sourceforge.net/) and visualized with Circos v0.51 [78].

## Supporting Information

Figure S1 Optical mapping of CP4 chromosome. Optical mapping of NcoI-digested genomic DNA isolated from strain CP4 was performed by OpGen, Inc. This technique is limited to the mapping of chromosomal fragments. The lower bar represents the optical map and the upper bar the in silico NcoI-digested pseudomolecule generated from the CP4 draft genome sequence. Lines linking the two bars represent NcoI-restriction sites matched by the optical mapping software. The black bar represents the sequence from the CP4 draft genome that did not align with the CP4 optical map.
(0.05 MB PDF)

Figure S2 F5/8 Type C Domain Protein Alignment and Peptide ID Matching. Multiple alignment of protein sequences from three paralogous F5/8 Type C domain proteins, two chromosomal (B and C) and one located in NELoc-1 (A), was used to generate a phylogenetic tree. Peptide sequences obtained from Kulkarni et al. [19] were compared against each of the three paralogs and amino acid differences are indicated in red.
(0.30 MB PDF)

Figure S3 Mapping of CP4 Solexa reads to plasmid sequences. CP4 Solexa reads were mapped to C. perfringens virulence plasmids A) pCPF5603, B) pCPF4969 and C) pCP8533etx using Maq, and the depth of coverage along the plasmid visualized with Circos. From inner to outer: ring 1, plasmid sequence with the conserved backbone region in red, variable region in blue, and the putative Tn916 region highlighted in orange; ring 2, open-reading frames, with forward in green and reverse in red; ring 3, coverage of CP4 Solexa reads, red peaks indicate >400× coverage.
(0.42 MB PDF)

Figure S4 Polymorphism maps of NE loci. Green vertical lines indicate approximate position of polymorphisms between the seven NE strains. The shortest green line represents a single nucleotide polymorphism (SNP) in one strain and the longest green line indicates a SNP in three strains.
(0.17 MB PDF)

Figure S5 Phylogenetic analysis of sequenced C. perfringens strains. The phylogenetic relationship among our eight sequenced isolates was assessed by (A) PFGE analysis of SmaI-digested genomic DNA and (B) sequence alignment of the whole proteome from each strain using CVtree. Additional eight publicly available C. perfringens genomes were also included for comparison.
(0.72 MB PDF)

Figure S6 Overlapping PCR analysis of NE locus 1. PCR products spanning the entire locus are represented by black bars and the PCR results for each strain tested are given below as follows: +.PCR product was of expected size; −, no PCR product produced. Where the PCR product did not match the expected size, the actual size is given.
(0.08 MB PDF)

Figure S7 Overlapping PCR analysis of NE locus 2. PCR products spanning the entire locus are represented by black bars and the PCR results for each strain tested are given below as follows: +.PCR product was of expected size; −, no PCR product produced.
(0.07 MB PDF)

Figure S8 Overlapping PCR analysis of NE locus 3. PCR products spanning the entire locus are represented by black bars and the PCR results for each strain tested are given below as follows: +.PCR product was of expected size; −, no PCR product produced.
(0.02 MB PDF)

Table S1 Primers used for PCR DIG labeling and sequencing.
(0.04 MB PDF)

Table S2 Primers used for overlapping PCR analysis of NELoc-1, -2 and -3.
(0.05 MB PDF)
