# Correction: Identification of Novel Pathogenicity Loci in *Clostridium perfringens* Strains That Cause Avian Necrotic Enteritis

**DOI:** 10.1371/annotation/501e5656-71ac-420f-a194-80141f6381e5

**Published:** 2010-06-24

**Authors:** Dion Lepp, Bryan Roxas, Valeria R. Parreira, Pradeep R. Marri, Everett L. Rosey, Joshua Gong, J. Glenn Songer, Gayatri Vedantam, John F. Prescott

The first three authors of this work, Dion Lepp, Bryan Roxas, and Valeria Parreira, should be considered as having contributed equally to this work. 

