# Supplementary figures and images for: Identification of Novel Pathogenicity Loci in Clostridium perfringens Strains That Cause Avian Necrotic Enteritis
Source: PLoS One. 2010 May 24;5(5):e10795. doi: 10.1371/journal.pone.0010795 (PMC2879425; doi:10.1371/journal.pone.0010795)

CP4 draft genome assembly

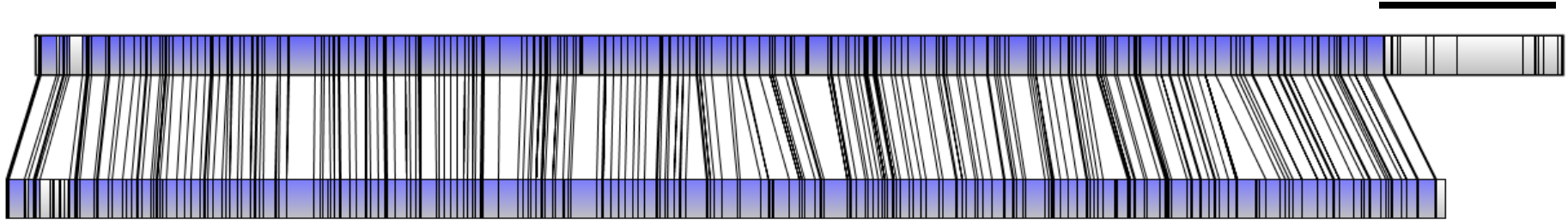

CP4 chromosomal optical map

Supplement: Figure S1 — Optical mapping of CP4 chromosome. Optical mapping of NcoI-digested genomic DNA isolated from strain CP4 was performed by OpGen, Inc. This technique is limited to the mapping of chromosomal fragments. The lower bar represents the optical map and the upper bar the in silico NcoI-digested pseudomolecule generated from the CP4 draft genome sequence. Lines linking the two bars represent NcoI-restriction sites matched by the optical mapping software. The black bar represents the sequence from the CP4 draft genome that did not align with the CP4 optical map. (0.05 MB PDF) [file pone.0010795.s001.pdf]

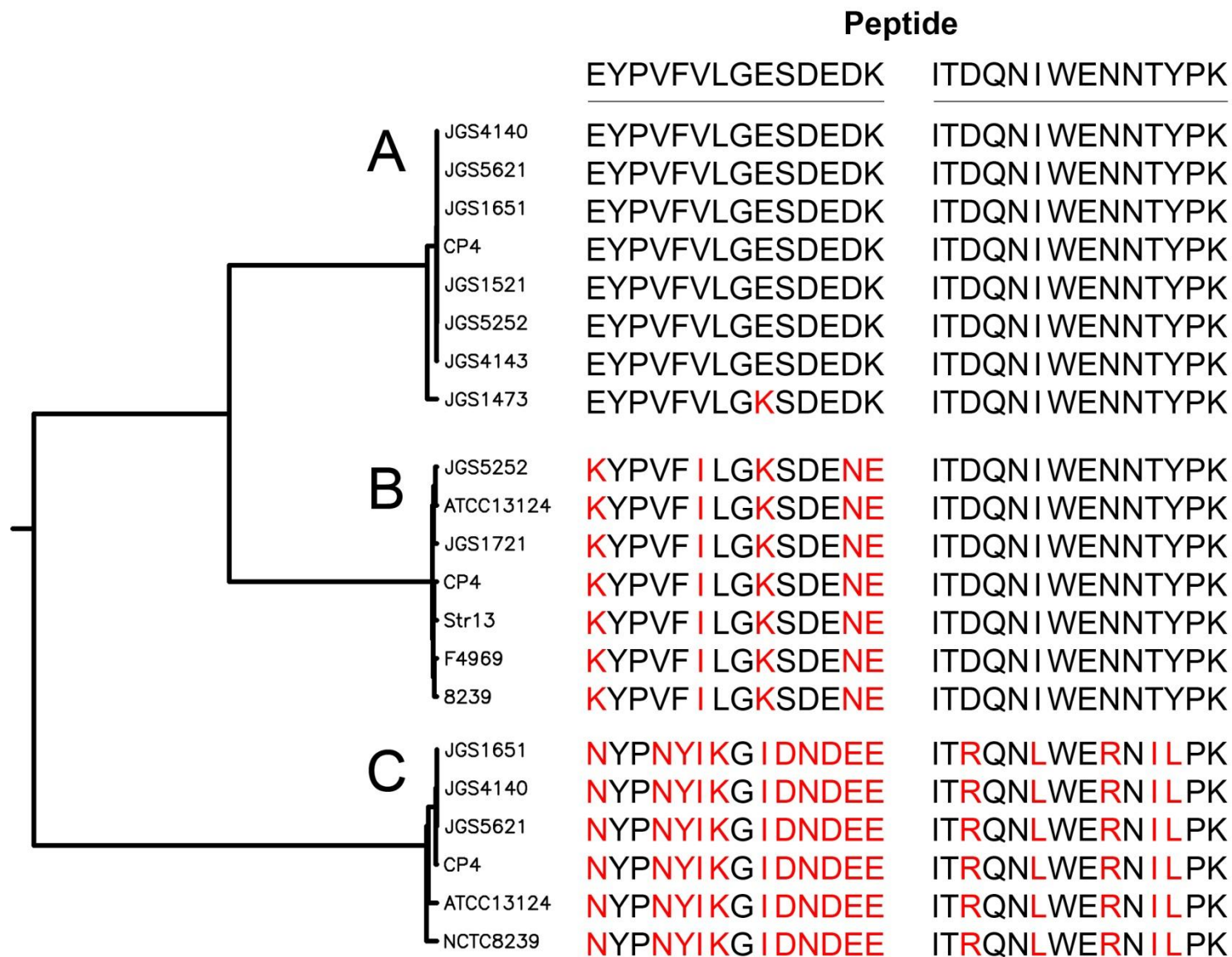

Supplement: Figure S2 — F5/8 Type C Domain Protein Alignment and Peptide ID Matching. Multiple alignment of protein sequences from three paralogous F5/8 Type C domain proteins, two chromosomal (B and C) and one located in NELoc-1 (A), was used to generate a phylogenetic tree. Peptide sequences obtained from Kulkarni et al. [19] were compared against each of the three paralogs and amino acid differences are indicated in red. (0.30 MB PDF) [file pone.0010795.s002.pdf]

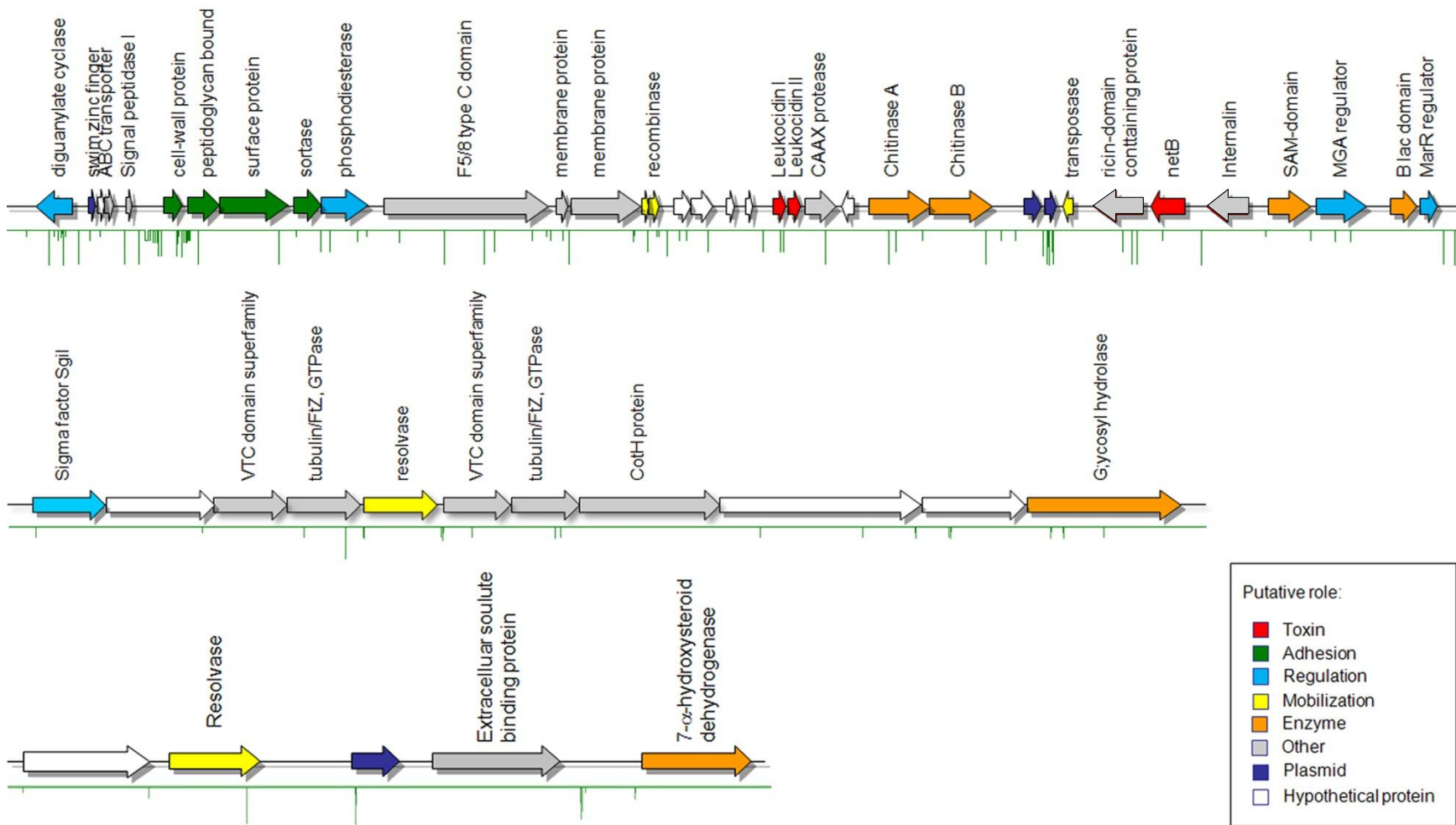

Supplement: Figure S4 — Polymorphism maps of NE loci. Green vertical lines indicate approximate position of polymorphisms between the seven NE strains. The shortest green line represents a single nucleotide polymorphism (SNP) in one strain and the longest green line indicates a SNP in three strains. (0.17 MB PDF) [file pone.0010795.s004.pdf]

A

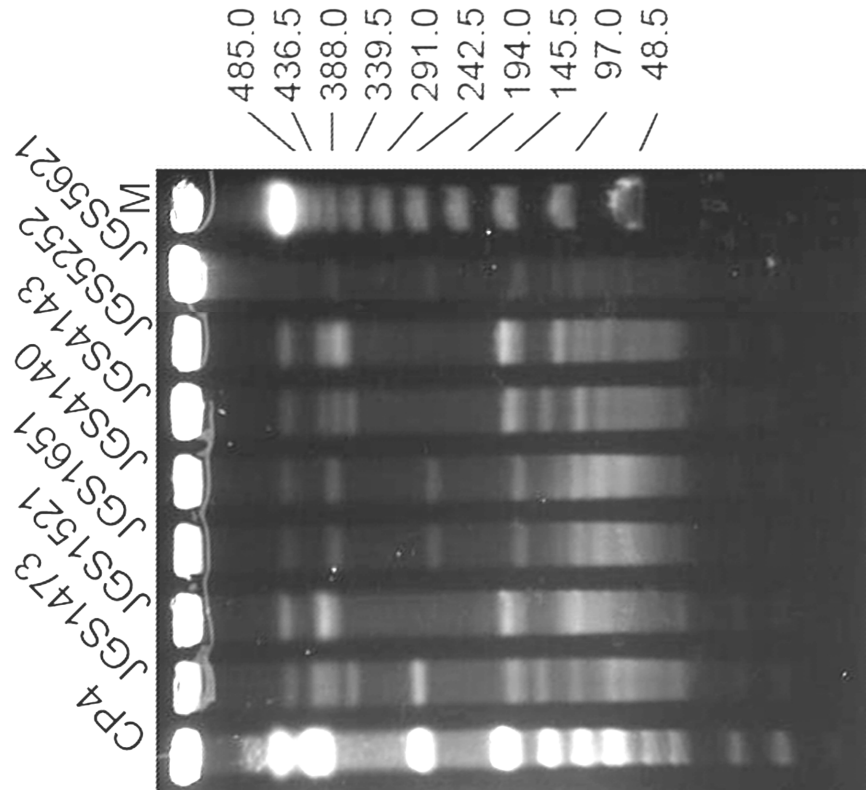

B

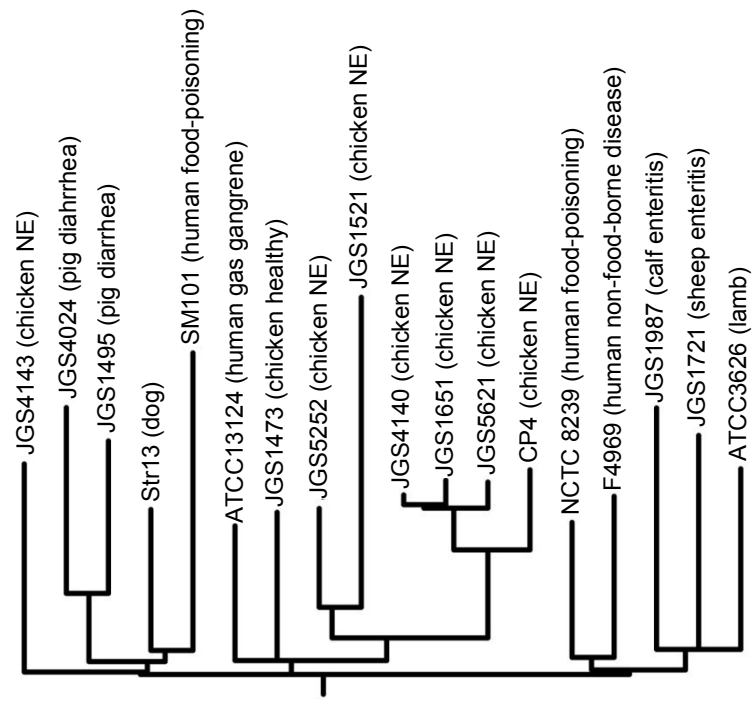

Supplement: Figure S5 — Phylogenetic analysis of sequenced C. perfringens strains. The phylogenetic relationship among our eight sequenced isolates was assessed by (A) PFGE analysis of SmaI-digested genomic DNA and (B) sequence alignment of the whole proteome from each strain using CVtree. Additional eight publicly available C. perfringens genomes were also included for comparison. (0.72 MB PDF) [file pone.0010795.s005.pdf]

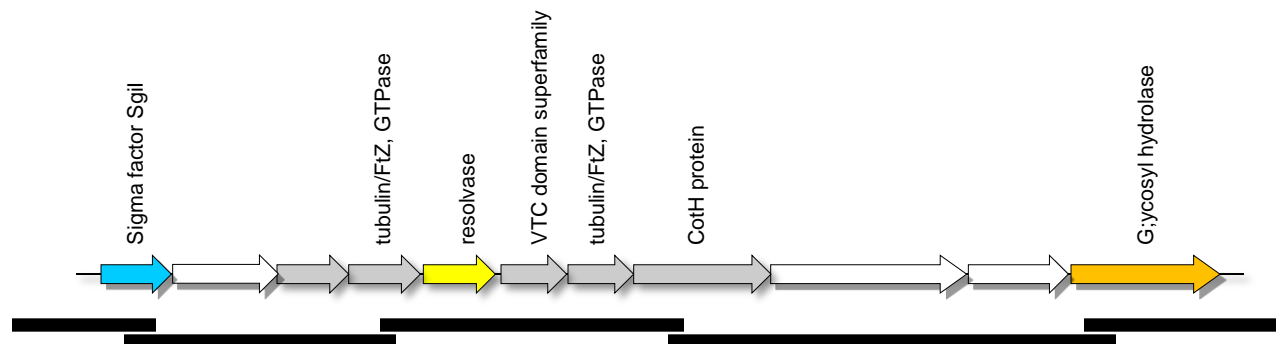

| Rxn No. | NEL2-link 5'<br>(928 bp) | NEL2-1<br>(2392 bp) | NEL2-2<br>(3037 bp) | NEL2-3<br>(4089 bp) | NEL2-link 3'<br>(1914 bp) |
|---------|--------------------------|---------------------|---------------------|---------------------|---------------------------|
| JGS1651 | +                        | +                   | +                   | +                   | +                         |
| JGS5252 | +                        | +                   | +                   | +                   | +                         |
| JGS5621 | +                        | +                   | +                   | +                   | +                         |
| JGS1521 | +                        | +                   | +                   | +                   | +                         |
| JGS4143 | +                        | +                   | +                   | +                   | +                         |
| JGS4140 | +                        | +                   | +                   | +                   | +                         |
| CP4     | +                        | +                   | +                   | +                   | +                         |
| CP1     | +                        | +                   | +                   | +                   | +                         |
| CP2     | +                        | +                   | +                   | +                   | +                         |
| CP3     | +                        | +                   | +                   | +                   | +                         |
| CP6     | +                        | +                   | +                   | +                   | +                         |
| JGS1473 | -                        | -                   | -                   | -                   | -                         |

Supplement: Figure S7 — Overlapping PCR analysis of NE locus 2. PCR products spanning the entire locus are represented by black bars and the PCR results for each strain tested are given below as follows: +.PCR product was of expected size; −, no PCR product produced. (0.07 MB PDF) [file pone.0010795.s007.pdf]

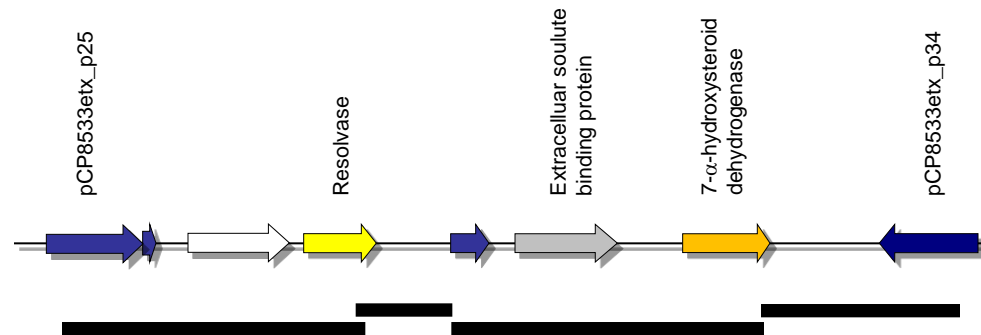

| Rxn No. | NEL3-1<br>(2.9 kb) | NEL3-2<br>(940 bp) | NEL3-3<br>(2.9 kb) | NEL3-4<br>(1.9 kb) |
|---------|--------------------|--------------------|--------------------|--------------------|
| JGS1651 | +                  | +                  | +                  | +                  |
| JGS5252 | +                  | +                  | +                  | +                  |
| JGS5621 | +                  | +                  | +                  | +                  |
| JGS1521 | +                  | +                  | +                  | +                  |
| JGS4143 | +                  | +                  | +                  | +                  |
| JGS4140 | +                  | +                  | +                  | +                  |
| CP4     | +                  | +                  | +                  | +                  |
| CP1     | +                  | +                  | +                  | +                  |
| CP2     | +                  | +                  | +                  | +                  |
| CP3     | +                  | +                  | +                  | +                  |
| CP6     | +                  | +                  | +                  | +                  |
| JGS1473 | -                  | -                  | -                  | -                  |

Supplement: Figure S8 — Overlapping PCR analysis of NE locus 3. PCR products spanning the entire locus are represented by black bars and the PCR results for each strain tested are given below as follows: +.PCR product was of expected size; −, no PCR product produced. (0.02 MB PDF) [file pone.0010795.s008.pdf]
