# Supplementary material for: Identification of Novel Pathogenicity Loci in Clostridium perfringens Strains That Cause Avian Necrotic Enteritis
Source: PLoS One. 2010 May 24;5(5):e10795. doi: 10.1371/journal.pone.0010795 (PMC2879425; doi:10.1371/journal.pone.0010795)
Supplement: Table S1 — Primers used for PCR DIG labeling and sequencing. (0.04 MB PDF) [file pone.0010795.s009.pdf]

## Table S1. Primers used for PCR DIG labeling and sequencing,

| PCR Reaction | Predict size (bp) | Primers | Primer sequences (5'-3') | Reference |
|---|---|---|---|---|
| DIG probe netB | 384 | AKP78<br>AKP79 | GCTGGTGCTGGAATAAATGC<br>TCGCCATTGAGTAGTTTCCC | Keyburn et al., 2008 |
| DIG probe hdhA | 387 | HDHA-F<br>HDHA-R | CAATTGTTACTGCAGCTTCAAGAGG<br>TAATTTTTACCGCCACCTTCATAATA | This study |
| Long Range PCR-5' NEL_1 | 8503 | SP-F<br>DCM-R | GTGCAGTTACTATTATAGGACC<br>TTGAAGCACTAAGGATTATAGA | This study |
| Long Range PCR-3' NEL_1 | 2677 | BLAC-F<br>LEXA-F | AACTACTTAATAGACACAGGAA<br>TACAGGATCAGTATCATATACC | This study |
| **CP1**pcr product 4214 bp | 3000 | WBRA-F<br>LKI-R | GCAGATTCACTTCCTGTAACAA<br>CAGTTCATATGTATGTGTTGAC | This study |
