# Supplementary material for: Identification of Novel Pathogenicity Loci in Clostridium perfringens Strains That Cause Avian Necrotic Enteritis
Source: PLoS One. 2010 May 24;5(5):e10795. doi: 10.1371/journal.pone.0010795 (PMC2879425; doi:10.1371/journal.pone.0010795)
Supplement: Table S2 — Primers used for overlapping PCR analysis of NELoc-1, -2 and -3. (0.05 MB PDF) [file pone.0010795.s010.pdf]

Table S2. Primers used for overlapping PCR analysis of NELoc-1, -2 and -3

PCR reaction	Predicted Size (bp)	Primers	Primer sequences
NEL1-Link5’	~9000	DCM-F	GTGAATCCTACAAGTCTCCAGC
		SIGP-F	GTGCAGTTACTATTATAGGACC
NEL1-1	3722	SIGPROT-R	CCAATACTGCATGTTACTTCTA
		03749-R	GTTGTTGTGTAATATATCTAGC
NEL1-2	4482	03749-F	GTATACTTCAGAAGGAACGGCT
		SORTA-R	TCTGCTTCACTAAGATCATTAT
NEL1-3	2737	SORTA-F	TATTGTCAGCTCATACAGGATT
		1281-R1	CTACTGTATTCCAAGCTGATCT
NEL1-4	4869	1281-F1	GGCTATACTTGTCGCATCATTA
		1281-R2	ACTTCATCATCAGTTGCATCTT
NEL1-5	5530	1281-F2	CAAGTCTTGAAGAAGTTATAGC
		WBRA-R	AGCTTCACCACTACCAATTGAT
NEL1-6	3000	WBRA-F	GCAGATTCACTTCCTGTAACAA
		LKI-R	CAGTTCATATGTATGTGTTGAC
NEL1-7	3564	LKII-F	TTATTCCTTCTTCACTTGTTCA
		CHIA-R	AAGGAGAAGCAGTAACAGCAGC
NEL1-8	2224	CHIA-F	TGTTGGAGGTTGGACAGGAACA
		CHIB-R	CTCCAAGCTCCAGCCATATCAT
NEL1-9	3942	CHIB-F	CAACAATAGCCGCAACTCCTTC
		tn1546-R	GAAGAACATTATACAGTCATAG
NEL1-10	3118	tn1546-F	TTGTCTAGAGTAAGAAGCTAAT
		AKP78	GCTGGTGCTGGAATAAATGC
NEL1-11	2760	AKP79	TCGCCATTGAGTAGTTTCCC
		INTER-F	CAACGTTAATAGTAGGTTCAAT
NEL1-12	4087	INTER-R	TCTCTAATGGACTTATATCTTC
		MPROT-R	TCATTATTATATGCTAATCCAA
NEL1-13	2926	MPROT-F	ATTGAATGGATGTATTATGCAG
		MAR-R	GTTATAATTGGAGTTAGTGTTC
NEL1-Link3’	2.7	BLAC-F	AACTACTTAATAGACACAGGAA
		LEXA-F	TACAGGATCAGTATCATATACC

NEL2-Link 5’	928	457-F	AGTTGCTGTTATGCTAGATAGG
		SigF-R	TGTAGTTACTTCTGAATCATGG
NEL2-1	2392	SigF-F	TCCTAAGCATAGAGATACAAGA
		FtZ-R	TCTGTACTAATTCCATAAGCAC
NEL2-2	3037	FtZ-F	ACAACTGTGCTTATGGAATTAG
		CotH-R	TTCTCCATTGGTGTCTATAACT
NEL2-3	4089	CotH-F	TTGATAGGCTTATGGAAGACAA
		Gh-R	GATATGTTGAAGCATTGATACC
NEL2-Link 3’	1914	Gh-F	AAGATCCAGAGGCCTATTAGAA
		469-R	TGCTGAAGCTACTAAGAATAGA
NEL3-Link5’	2.9	orf53-F1	CCAACAACTCCCATAAAAGATG
		res-R1	GCAGTTAATTCTCCGTTTTTCC
NEL3-1	0.94	1852_R2	CATCCAAGGCTCATCACTCC
		1852-F2	GAAGTGCCATCTAATTTTGAAGAAA
NEL3-2	2.9	1852_F1	GGAGTGATGAGCCTTGGATG
		1852_R1	AAGAGCAAATCCTCCAGCAA
NEL3-Link3’	1.9	5603_56-F1	TCCTACATGGACAATTGCTGA
		4143_3417-F1	TTGCTGGAGGATTTGCTCTT
