# Supplementary material for: Identification of Novel Pathogenicity Loci in Clostridium perfringens Strains That Cause Avian Necrotic Enteritis
Source: PLoS One. 2010 May 24;5(5):e10795. doi: 10.1371/journal.pone.0010795 (PMC2879425; doi:10.1371/journal.pone.0010795)
Supplement: Figure S3 — Mapping of CP4 Solexa reads to plasmid sequences. CP4 Solexa reads were mapped to C. perfringens virulence plasmids A) pCPF5603, B) pCPF4969 and C) pCP8533etx using Maq, and the depth of coverage along the plasmid visualized with Circos. From inner to outer: ring 1, plasmid sequence with the conserved backbone region in red, variable region in blue, and the putative Tn916 region highlighted in orange; ring 2, open-reading frames, with forward in green and reverse in red; ring 3, coverage of CP4 Solexa reads, red peaks indicate >400× coverage. (0.42 MB PDF) [file pone.0010795.s003.pdf]

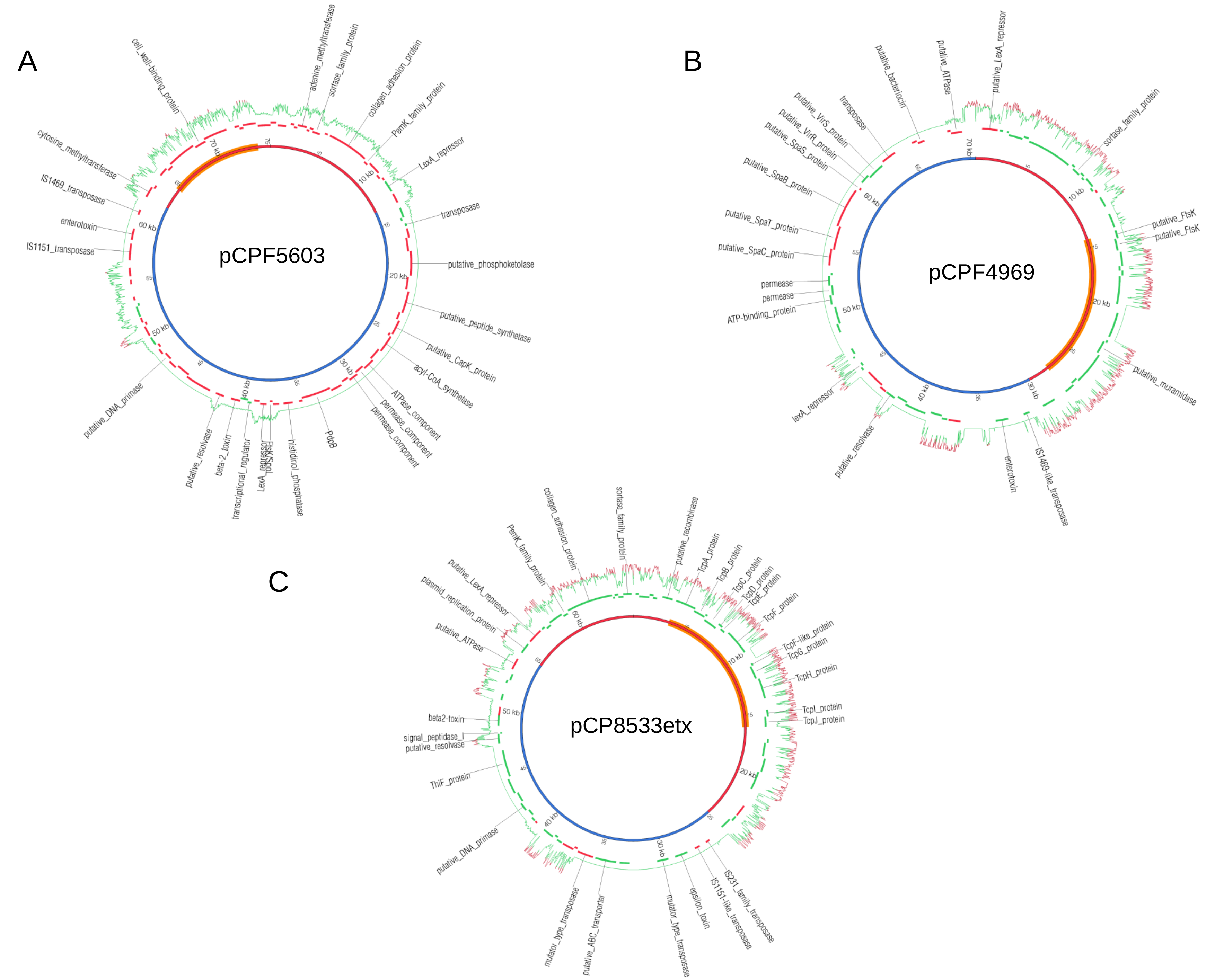

A
pCPF5603
cell_wall-binding_protein
adenine_methyltransferase
sortase_family_protein
collagen_adhesion_protein
PemK_family_protein
LexA_repressor
transposase
putative_phosphoketolase
putative_peptide_synthetase
putative_CapK_protein
acyl-CoA_synthetase
ATPase_component
permease_component
permease_component
PcpB
histidinol_phosphatase
transcriptional_regulator
beta-2_toxin
putative_resolvase
putative_DNA_primase
IS1151_transposase
enterotoxin
IS1469_transposase
cytosine_methyltransferase
B
pCPF4969
putative_bacteriocin
putative_ATPase
putative_LexA_repressor
sortase_family_protein
transposase
putative_VirS_protein
putative_VirR_protein
putative_SpaS_protein
putative_SpaB_protein
putative_SpaT_protein
putative_SpaC_protein
permease
permease
ATP-binding_protein
putative_FtsK
putative_FtsK
putative_muramidase
lexA_repressor
putative_resolvase
enterotoxin
IS1469-like_transposase
C
pCP8533etx
collagen_adhesion_protein
sortase_family_protein
putative_recombinase
PemK_family_protein
putative_LexA_repressor
plasmid_replication_protein
putative_ATPase
TcpA_protein
TcpB_protein
TcpC_protein
TcpD_protein
TcpE_protein
TcpF_protein
TcpF-like_protein
TcpG_protein
TcpH_protein
TcpI_protein
TcpJ_protein
beta2-toxin
signal_peptidase_I
putative_resolvase
ThiF_protein
putative_DNA_primase
mutator_type_transposase
putative_ABC_transporter
mutator_type_transposase
epsilon_toxin
IS1151-like_transposase
IS231_family_transposase
