# Supplementary material for: Identification of Novel Pathogenicity Loci in Clostridium perfringens Strains That Cause Avian Necrotic Enteritis
Source: PLoS One. 2010 May 24;5(5):e10795. doi: 10.1371/journal.pone.0010795 (PMC2879425; doi:10.1371/journal.pone.0010795)
Supplement: Figure S6 — Overlapping PCR analysis of NE locus 1. PCR products spanning the entire locus are represented by black bars and the PCR results for each strain tested are given below as follows: +.PCR product was of expected size; −, no PCR product produced. Where the PCR product did not match the expected size, the actual size is given. (0.08 MB PDF) [file pone.0010795.s006.pdf]

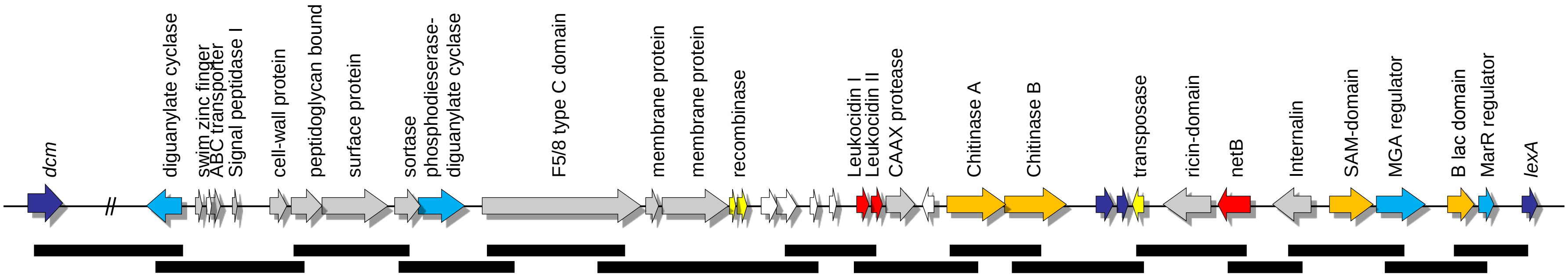

Rxn No.	NEL1-link 5' (9 kb)	NEL1-1 (4.1 kb)	NEL1-2 (4.5 kb)	NEL1-3 (2.7 kb)	NEL1-4 (4.9 kb)	NEL1-5 (5.5 kb)	NEL1-6 (3 kb)	NEL1-7 (3.6 kb)	NEL1-8 (2.2 kb)	NEL1-9 (3.9 kb)	NEL1-10 (3.1 kb)	NEL1-11 (2.8 kb)	NEL1-12 (4.1 kb)	NEL1-13 (2.9 kb)	NEL1-link 3' (2.7 kb)
JGS1651	+[1]	+	+	+	+	+	+	+	+	+	+	+	+	+	+
JGS5252	+	+	+	+	+	+	+	+	+	+	+	+	+	+	+
JGS5621	+	+	+	+	+	+	+	+	+	+	+	+	+	+	+
JGS1521	+	+	+	+	+	+	+	+	+	+	+	+	+	+	+
JGS4143	+	+	+	+	+	+	+	+	+	+	+	+	+	+	+
JGS4140	+	+	+	+	+	+	+	+	+	+	+	+	+	+	+
CP4	+	+	+	+	+	+	+	+	+	+	+	+	+	+	+
CP1	+	+	+	+	+	+	4.2	+	+	+	+	+	+	+	+
CP2	+	+	+	+	+	+	-	-	-	-	+	+	+	+	+
CP3	+	+	+	+	+	+	+	+	+	+	+	+	+	+	+
CP6	-	-	-	-	-	-	-	+	+	-	-	-	-	-	-
JGS1473	-	-	-	-	+	-	-	-	-	-	-	-	-	-	-
